# Characterization of emerging Newcastle disease virus isolates in China

**DOI:** 10.1186/s12985-015-0351-z

**Published:** 2015-08-07

**Authors:** Jing-Yu Wang, Wan-Hua Liu, Juan-Juan Ren, Pan Tang, Ning Wu, Hung-Yi Wu, Ching-Dong Ching, Hung-Jen Liu

**Affiliations:** College of Veterinary Medicine, Northwest A & F University, Yangling, 712100 China; Institute of Molecular Biology, National Chung Hsing University, Taichung, 402 Taiwan; Agricultural Biotechnology Center, National Chung Hsing University, Taichung, 402 Taiwan; Rong Hsing Research Center for Translational Medicine, National Chung Hsing University, Taichung, 402 Taiwan; Department of Veterinary Medicine, National Pingtung University of Science and Technology, Pingtung, 912 Taiwan

**Keywords:** Newcastle disease virus, F gene, HN gene, Phylogenetic analysis, Cross-protection experiment

## Abstract

**Background:**

Newcastle disease (ND) is a devastating worldwide disease of poultry characterized by increased respiration, circulatory disturbances, hemorrhagic enteritis, and nervous signs. Sequence analysis shows several amino acid residue substitutions at neutralizing epitopes on the F and HN proteins of recent Shaanxi strains. Both Cross protection and cross serum neutralization tests revealed that the traditional vaccine strains were unable to provide full protection for the flocks.

**Methods:**

To better understand the epidemiology of Newcastle disease outbreak, a portion of the F gene and the full-length HN gene were amplified from Shaanxi isolates by reverse transcription-polymerase chain reaction (RT-PCR) and then conducted sequence and phylogenetic analyzes. In pathogenicity analysis**,** both high intra-cerebral pathogenicity index (ICPI) and mean death time (MDT) tests of chicken embryo were carried out. Furthermore, a cross-protection experiment in which specific-pathogen-free chickens vaccinated with a LaSota vaccine strain were challenged by the recent Shaanxi strain was also performed.

**Results:**

Nine Newcastle disease (ND) virus (NDV) isolates which were recovered from ND outbreaks in chicken flocks in China were genotypically and pathotypically characterized. Amino acid sequence analysis revealed that all the recent Shaanxi-isolated NDVs have ^112^R-R-Q-K-R-F^117^ for the C-terminus of the F2 protein and exhibit high ICPI and MDT of chicken embryos, suggesting that they were all classified as velogenic type of NDVs. Phylogenetic analysis of these isolates showed that they belong to subgenotype VIId that have been implicated in the recent outbreaks in northwestern China. The percentage of amino acid sequence identity of F protein between recent Shaanxi stains and five vaccine strains was in the range of 81.9 %–88.1 %, while the percentage of amino acid sequence identity of HN protein between recent Shaanxi strains and vaccine strains was in the range of 87.4 %–91.2 %. Furthermore, a number of amino acid residue substitutions at neutralizing epitopes on the F and HN proteins of these isolates were observed, which may lead to the change of antibody recognition and neutralization capacity. A cross-protection experiment indicated that specific-pathogen-free chickens vaccinated with a LaSota vaccine strain was not capable of providing full protection for the flocks that were challenged by the recent Shaanxi strain.

**Conclusions:**

Taken together, our findings reveal that recent Shannxi NDVstrains exhibit antigenic variations that could be responsible for recent outbreaks of NDVs in northwestern China.

## Background

Newcastle disease is a highly contagious disease, which caused by the virulent strains of Newcastle disease virus (NDV), and causes great economic loss to the global poultry industry. In addition to poultry species, at least 250 species of birds can be naturally or experimentally infected with NDV [1]. The major signs of the disease include respiratory distress, diarrhea, circulatory disturbances, and central nervous system impairment [1–3]. Although NDV has only 1 serotype, ND outbreaks still occur frequently in flocks vaccinated with NDV live vaccines (e.g. Lasota and Clone 30) and inactivated vaccines in China. Immune failure is considered to be related to the genetic variation of NDV epidemic strains [3–6].

On the basis of restriction fragment length polymorphism (RFLP) analysis and partial nucleotide sequencing of the cleavage recognition sequence of the F gene, NDV strains are grouped into at least 10 genogroups, among which genogroup VII can be further divided into several sub-genotypes [5, 7, 8]. The currently circulating NDVs in the worldwide exhibit multiple genotypes and high genetic diversity. According to pathogenicity assays in specific pathogen free (SPF) chickens, NDVs have been categorized into velogenic, mesogenic, and lentogenic pathotypes. The F protein mediates the fusion of viral and cellular membranes during penetration and spread between infected and adjacent cells. The virulence of NDV can be predicted by analyzing the cleavage site sequence of F protein [1, 9, 10]. By comparing the amino acid residues of the C-terminal HN extension of NDV, they were divided into at least 3 groups, including the numbers of amino acid residues of 571, 577 and 616 [11, 12]. The viral HN protein mediates attachment to sialic acid-containing receptor(s) through its neuraminidase (NA) activity. HN is a type II membrane glycoprotein, which exists on the surface of virions and infected cells as a tetrameric spike. The ectodomain of the HN glycoprotein consists of a membrane-proximal, stalk-like segment supporting a terminal globular domain. The antigenic, receptor recognition, and NA active sites all reside in the latter [13]. Previous studies have suggested that there are at least five antigenic sites related to epitopes on the HN protein of NDV, which are related to antibody recognition [13, 14].

In this study, nine NDV strains have been isolated from NDV-infected chickens in Shaanxi province in China. To investigate the epidemiology of Newcastle disease outbreak, a portion of the F gene and the full-length HN gene were amplified from these isolates by reverse transcription-polymerase chain reaction (RT-PCR) and then conducted sequence analysis. The nucleotide sequences of the F and HN genes of NDVs from recent Shaanxi isolates were compared with published sequences to clarify the evolution, epidemiology and their pathotypes of NDV. Our findings reveal that all Shaanxi NDV strains belong to genotype VIId. Sequence analysis showed several amino acid residue substitutions at neutralizing epitopes on the F and HN proteins of recent isolated Shaanxi strains. Furthermore, cross protection and cross serum neutralization tests suggested that the traditional vaccine strains such as LaSota were unable to provide full protection for the flocks.

## Results

### Pathogenicity analysis and RT-PCR

Nine NDV strains were isolated from NDV-infected chickens during 2011 and named NDV/Chicken/TC/1/2011, NDV/Chicken/TC/2/2011, NDV/Chicken/ TC/3 /2011, NDV/Chicken/TC/4/2011, NDV/Chicken/TC/5/2011, NDV/Chicken/ TC/6 /2011, NDV/Chicken/TC/7/2011, NDV/Chicken/TC/8/2011, and NDV/Chicken /TC/9/2011 (Table 1). All Shaanxi strains of NDV with MDT of 38–50 h and with ICPI of 1.67-1.78 (Table 1) were classified as virulent NDVs. By using the primers F1 and F2, the fragments of the F gene were amplified from all Shaanxi NDV isolates with the expected size of 957 bp in length. By using the primer pairs (HN1-HN2 and HN3-HN4), 2 PCR products (1002 and 1338 bp) covered the full-length HN gene (1716 bp) could be amplified from all Shaanxi NDV isolates (data not shown).

**Table 1:** Characterization of NDV strains used in this study

NDV strain	COU	HO	Year	PT	Cleavage site112–117	GT	ICPI	F No.	HN No.
Clone 30	USA	CK	1999	V	GRQGRL	II	NA	Y18898	Y18898
B1	USA	CK	2000	L	GRQGRL	II	0.13	AF309418	AF309418
LaSota	USA	CK	1950	L	GRQGRL	II	0.31	JF950510	JF950510
Mukteswar	CH	CK	2010	L	RRQRRF	III	NA	JF950509	JF950509
Herts/33	NL	CK	2004	V	RRQRRF	IV	1.99	AY741404	AY741404
NL/152608/93	NL	CK	1993	V	RRQKRF	VIIa	NA	JN986837	JN986837
chicken/Kudus/018/10	INA	CK	2010	V	RRQKRF	VIIa	NA	HQ697260	HQ697260
chicken/Sweden/97	SE	CK	1997	V	RRQRRF	VIIb	NA	GU585905	GU585905
Sterna/Astr/2755/2001	RUS	CK	2001	V	RRQRRF	VIIb	NA	AY865652	AY865652
TW-99-175	TW	CK	1999	V	RRQKRF	VIIc	1.73	EU604057	EU579448
XJ-2/97	CH	CK	1997	V	RRQKRF	VIId	1.94	AF458011	AF458011
JS/1/97/Go	CH	GO	1997	V	RRQRRF	VIId	1.84	AF456435	AF456429
BP01	CH	PE	1999	V	RRQKRF	VIId	NA	JN599167	JN599167
JS/5/06/Go	CH	GO	2006	V	RRQKRF	VIId	NA	EF211811	EF211811
HA-14-07-Ch	CH	CK	2007	V	RRQKRF	VIId	NA	GQ245787	GQ245787
JSD0812	CH	DK	2008	V	RRQKRF	VIId	NA	GQ849007	GQ849007
sh09	CH	CK	2009	V	RRQKRF	VIId	NA	GU124591	GU124592
chicken/China/JSX1/2010	CH	CK	2010	V	RRQKRF	VIId	NA	JX519467	JX519467
JS-12-11-Ch	CH	CK	2011	V	RRQKRF	VIId	NA	JQ013866	JQ013842
Ch/SD672/12	CH	CK	2012	V	RRQKRF	VIId	NA	KC020114	JX914489
NDV/Chicken/TC/1/2011	CH	CK	2011	V	RRQKRF	VIId	1.78	KC020314	KC020305
NDV/Chicken/TC/2/2011	CH	CK	2011	V	RRQKRF	VIId	1.76	KC020315	KC020306
NDV/Chicken/TC/3/2011	CH	CK	2011	V	RRQKRF	VIId	1.73	KC020316	KC020307
NDV/Chicken/TC/4/2011	CH	CK	2011	V	RRQKRF	VIId	1.75	KC020317	KC020308
NDV/Chicken/TC/5/2011	CH	CK	2011	V	RRQKRF	VIId	1.67	KC020318	KC020309
NDV/Chicken/TC/6/2011	CH	CK	2011	V	RRQKRF	VIId	1.68	KC020319	KC020310
NDV/Chicken/TC/7/2011	CH	CK	2011	V	RRQKRF	VIId	1.67	KC020320	KC020311
NDV/Chicken/TC/8/2011	CH	CK	2011	V	RRQKRF	VIId	1.70	KC020321	KC020312
NDV/Chicken/TC/9/2011	CH	CK	2011	V	RRQKRF	VIId	1.69	KC020322	KC020313
PX2/03	CH	DK	2003	V	RRQKRF	VIId	NA	EF175145	EF175145
Hebei/01/2012	CH	CK	2012	V	RRQKRF	VIId	1.73	KC542914	KC542914
Shandong/02/2012	CH	CK	2012	V	RRQKRF	VIId	NA	KC542913	KC542913
YZCQ/Liaoning/08	CH	CK	2008	V	RRQKRF	VIId	NA	FJ608351	FJ608369
Beijing/01/2012	CH	CK	2012	V	RRQKRF	VIId	1.91	KC542911	KC542911
SD-02-11-Ch	CH	CK	2011	V	RRQKRF	VIId	NA	JQ013878	JQ013849
Ch/SD754/12	CH	CK	2012	V	RRQKRF	VIId	NA	JX840452	JX840450
Ch/SD755/12	CH	CK	2012	V	RRQKRF	VIId	NA	JX840453	JX840451
Ch/SD758/12	CH	CK	2012	V	RRQKRF	VIId	NA	JX840455	JX683723
Ch/SD01/13	CH	CK	2013	V	RRQKRF	VIId	NA	KF055273	KF055274
TW/99-156B	TW	CK	1999	V	RRQKRF	VIIe	1.79	AF234031	AF400504
TW-02-301	TW	CK	2002	V	RRQKRF	VIIe	1.75	DQ898538	EU526302
TW-02-302	TW	CK	2002	V	RRQKRF	VIIe	1.75	DQ898537	EU526303
TW-04-CB8	TW	CK	2004	V	RRQKRF	VIIe	1.75	DQ898528	AB531987
TW/08-02	TW	CK	2008	V	RRQKRF	VIIe	NA	AB512614	AB531988

NL/152608/93: APMV-1/chicken/NL/152608/93; Hebei/01/2012: chicken/China/ Hebei/01/2012; Shandong/02/2012: chicken/China/ Shandong/02/2012; Beijing/01/2012: chicken/China/ Beijing/01/2012. CH: China; NL: Netherlands; INA: Indonesia; SE: Sweden; RUS: Russia; TW: Taiwan; GO: Goose; CK: Chicken; DK: Duck; PE: Penguin; COU: Country; HO: Host; PT: Pathotype; GT: Genotype; F no.: F gene accession no.; HN no.: HN gene accession no.; NA: not available

Clone 30, LaSota, Mukteswar and B1 strains are attenuated vaccine strains that widely used in the China and world; the Herts/33 are inactivated vaccine strains that used Taiwan

### Nucleotide and amino acid sequence analysis of the F gene of NDVs

All the recent Shaanxi strains contain the amino acid sequences ^112^R-R-Q-K-R-F^117^ for the C-terminus of the F2 protein, indicating that they are velogenic type of NDV. Furthermore, all the recent Shaanxi strains possess ^101^ K and ^121^ V (Table 2), a characteristic of genotype VII viruses reported previously [15].

**Table 2:** Alignment of deduced amino acid sequences of the F protein of NDV

strains	Amino acid residues																																											
Clone 30	M	G	S	R	P	S	T	K	N	P	A	P	M	M	L	T	I	R	V	A	L	V	L	S	C	I	C	P	A	N	S	I	D	G	R	P	L	A	A	A	40			
B1	.	.	.	.	.	F	.	.	.	.	.	.	.	.	.	.	.	.	.	.	.	.	.	.	.	.	.	L	.	.	.	.	.	.	.	.	F	.	.	.				
LaSota	.	.	.	.	.	.	.	.	.	.	.	.	.	.	.	.	.	.	.	.	.	.	.	.	.	.	.	L	.	.	.	.	.	.	.	.	.	.	.	.				
Mukteswar	.	.	P	.	S	.	.	R	I	.	V	.	L	.	.	.	.	.	I	T	.	A	.	.	V	R	Q	T	V	S	.	L	.	.	.	.	.	.	.	.				
Herts/33	.	.	.	.	S	.	.	R	I	.	V	.	P	.	.	I	.	.	I	V	.	T	.	.	.	.	Q	T	V	S	.	L	.	.	.	.	.	.	.	.				
NL/152608/93	.	.	.	K	.	.	.	R	T	.	V	.	L	.	.	I	T	.	I	M	.	I	S	.	.	.	.	L	V	S	.	L	.	.	.	.	.	.	.	.				
chicken/Kudus/018/10	.	.	.	K	.	.	.	R	I	.	V	.	L	.	.	I	T	.	I	M	.	I	.	.	Y	.	.	L	V	S	.	L	.	.	.	.	.	.	.	.				
chicken/Sweden/97	.	.	.	K	.	.	.	R	I	.	V	.	L	.	.	I	T	.	.	M	.	I	.	.	.	.	R	S	I	S	.	L	.	.	.	.	.	.	.	.				
Sterna/Astr/2755/2001	.	.	.	K	.	.	.	R	I	L	V	.	L	.	.	I	T	.	.	M	.	I	.	.	.	V	R	S	T	S	.	L	.	.	.	.	.	.	.	.				
TW-99-175	.	.	.	.	S	F	.	R	I	.	.	.	L	.	.	I	T	.	I	M	.	I	.	.	.	.	.	L	T	S	.	L	.	.	.	.	.	.	.	.				
XJ-2/97				K				R	I				L			I	T		I	M		I					R	L	T	S		L												
JS/1/97/Go	.	.	P	K	S	.	.	N	V	.	.	.	L	.	.	.	V	.	I	.	.	A	.	.	.	V	R	L	T	.	.	L	.	.	.	.	.	.	.	.				
BP01	.	.	.	K	.	.	.	R	I	.	.	.	L	.	.	I	T	.	I	M	.	I	.	G	.	.	R	.	T	S	.	L	.	.	.	.	.	.	.	.				
Guangxi9/2003	.	.	.	K	.	.	.	R	S	.	.	.	L	.	.	I	.	.	I	M	.	I	.	.	.	.	R	.	T	S	.	L	.	.	.	.	.	.	.	.				
JS/9/05/Go	.	.	P	.	S	.	.	R	I	.	V	.	L	.	.	T	.	.	I	T	.	A	.	.	V	R	Q	T	V	S	.	L	.	.	.	.	.	.	.	.				
HA-14-07-Ch	.	.	.	K	.	.	.	R	I	.	.	.	L	.	.	V	T	.	I	M	.	I	.	G	.	.	R	.	T	S	.	L	.	.	.	.	.	.	.	.				
JSD0812	.	.	.	K	.	.	.	R	I	.	.	.	L	.	.	I	T	.	T	M	.	I	.	G	.	.	.	.	T	S	.	L	.	.	.	.	.	.	.	.				
sh09	.	.	.	.	.	.	.	R	I	.	.	.	L	.	.	I	T	Q	I	M	.	I	.	.	.	.	.	.	T	S	.	L	.	.	.	.	.	.	.	.				
chicken/China/JSX1/2010	.	.	.	K	S	.	.	R	I	.	.	.	P	.	.	V	T	.	I	M	.	I	.	G	.	.	R	.	T	S	.	L	.	.	.	.	.	.	.	.				
JS-12-11-Ch	.	.	.	K	L	.	.	R	I	.	.	.	L	.	.	I	T	.	I	I	.	I	.	.	.	.	R	.	T	S	.	L	.	.	.	.	.	.	.	.				
NDV/Chicken/TC/1/2011	.	.	.	K	.	.	.	R	I	.	.	.	P	V	.	V	T	.	.	M	.	T	.	.	.	.	R	.	T	S	.	L	.	.	.	.	.	.	.	.				
NDV/Chicken/TC/2/2011	.	.	.	K	.	.	.	R	I	.	.	.	P	V	.	V	T	.	.	M	.	T	.	.	.	.	R	.	T	S	.	L	.	.	.	.	.	.	.	.				
NDV/Chicken/TC/3/2011	.	.	.	K	.	.	.	R	I	.	.	.	P	V	.	V	T	.	.	M	.	T	.	.	.	.	R	.	T	S	.	L	.	.	.	.	.	.	.	.				
NDV/Chicken/TC/4/2011	.	.	.	K	.	.	.	R	I	.	.	.	P	V	.	V	T	.	.	M	.	T	.	.	.	.	R	.	T	S	.	L	.	.	.	.	.	.	.	.				
NDV/Chicken/TC/5/2011	.	.	.	K	.	.	.	R	I	.	.	.	P	V	.	V	T	.	.	M	.	T	.	.	.	.	R	.	T	S	.	L	.	.	.	.	.	.	.	.				
NDV/Chicken/TC/6/2011	.	.	.	K	.	.	.	R	I	.	.	.	P	V	.	V	T	.	.	M	.	T	.	.	.	.	R	.	T	S	.	L	.	.	.	.	.	.	.	.				
NDV/Chicken/TC/7/2011	.	.	.	K	.	.	.	R	I	.	.	.	P	V	.	V	T	.	.	M	.	T	.	.	.	.	R	.	T	S	.	L	.	.	.	.	.	.	.	.				
NDV/Chicken/TC/8/2011	.	.	.	K	.	.	.	R	I	.	.	.	P	V	.	V	T	.	.	M	.	T	.	.	.	.	R	.	T	S	.	L	.	.	.	.	.	.	.	.				
NDV/Chicken/TC/9/2011	.	.	.	K	.	.	.	R	I	.	.	.	P	V	.	V	T	.	.	M	.	T	.	.	.	.	R	.	T	S	.	L	.	.	.	.	.	.	.	.				
PX2/03	.	.	.	K	.	.	.	R	V	.	.	.	L	.	.	I	T	.	I	M	.	I	.	G	.	.	.	S	T	S	.	L	.	.	.	.	.	.	.	.				
Chicken/China/Hebei/02/2012	.	.	.	K	.	.	.	R	I	.	.	.	L	.	.	I	T	.	I	M	.	I	.	.	.	.	R	L	T	S	.	L	.	.	.	.	.	.	.	.				
Shangdong/02/2012	.	.	.	K	.	.	.	R	I	.	.	.	L	.	.	I	T	.	I	M	.	T	.	.	.	.	R	L	T	S	.	L	.	.	.	.	.	.	.	.				
YZCQ/Liaoning/08	.	.	.	K	.	.	.	R	I	.	.	.	L	.	.	I	T	.	I	M	.	I	.	D	.	.	R	.	T	S	.	L	.	.	.	.	.	.	.	.				
Beijing/01/2012	.	.	.	K	.	.	.	.	I	.	V	.	L	.	.	I	T	.	I	M	.	I	.	N	.	.	R	L	T	S	.	L	.	.	.	.	.	.	.	.				
SD-02-11-Ch	.	.	.	K	L	.	.	R	I	.	.	.	L	.	.	I	T	.	I	M	.	T	.	.	.	.	R	L	T	S	.	L	.	.	.	.	.	.	.	.				
Ch/SD672/12	.	.	.	K	.	.	.	R	I	.	.	.	L	.	.	I	T	.	I	M	.	T	.	.	.	.	R	.	T	S	.	L	.	.	.	.	.	.	.	.				
Ch/SD754/12	.	.	.	K	.	.	.	R	I	.	.	.	L	.	.	I	T	.	I	M	.	T	.	.	.	.	R	L	T	S	.	L	.	.	.	.	.	.	.	.				
Ch/SD755/12	.	.	.	K	.	.	.	R	I	.	.	.	L	.	.	I	T	.	I	M	.	T	.	.	.	.	R	L	T	S	.	L	.	.	.	.	.	.	.	.				
Ch/SD758/12	.	.	.	K	.	.	.	R	I	.	.	.	L	.	.	I	T	.	I	M	.	T	.	.	.	.	R	L	T	S	.	L	.	.	.	.	.	.	.	.				
Ch/SD01/13	.	.	.	K	.	.	.	R	I	.	.	.	L	.	.	I	T	.	I	L	.	I	.	N	.	.	R	L	T	S	.	L	.	.	.	.	.	.	.	.				
TW/99-156B	.	.	.	.	S	.	.	R	I	.	.	.	L	.	.	.	T	Q	I	M	.	T	.	.	.	.	R	L	T	S	.	L	.	.	.	.	.	.	.	.				
TW-02-301	.	.	P	.	S	.	.	R	I	.	.	.	L	.	.	I	T	.	I	M	.	T	F	.	.	.	R	L	T	S	.	L	.	.	.	.	.	.	.	.				
TW-02-302	.	.	.	.	S	.	.	R	I	.	.	.	L	.	.	I	T	.	I	M	.	T	F	.	.	.	R	L	T	S	.	L	.	.	.	.	.	.	.	.				
TW-04-CB8	.	.	P	.	S	.	.	R	I	.	.	.	L	.	.	I	T	.	I	M	.	T	.	.	.	.	R	L	T	S	.	L	.	.	.	.	.	.	.	.				
TW/08-02	.	.	P	Q	T	F	.	R	I	.	.	.	L	.	.	I	T	.	I	M	.	T	F	.	.	T	R	L	.	S	.	L	.	.	.	.	.	.	.	.				
strains	Amino acid residues																																											
Clone 30	G	I	V	V	T	G	D	K	A	V	N	I	Y	T	S	S	Q	T	G	S	I	I	V	K	L	L	P	N	L	P	K	D	K	E	A	C	A	K	A	P	80			
B1	.	.	.	.	.	.	.	.	.	.	.	.	.	.	.	.	.	.	.	.	.	.	.	.	.	.	.	.	.	.	.	.	.	.	.	.	.	.	.	.				
LaSota	.	.	.	.	.	.	.	.	.	.	.	.	.	.	.	.	.	.	.	.	.	.	.	.	.	.	.	.	.	.	.	.	.	.	.	.	.	.	.	.				
Mukteswar	.	.	.	.	.	.	.	.	.	.	.	.	.	.	.	.	.	.	.	.	.	.	.	.	.	.	.	.	M	.	.	.	.	.	.	.	.	.	.	.				
Herts/33	.	.	.	.	.	.	.	.	.	.	.	.	.	.	.	.	.	.	.	.	.	.	.	.	.	.	.	.	M	.	.	.	.	.	.	.	.	.	.	.				
NL/152608/93	.	.	.	.	.	.	.	.	.	.	.	.	.	.	.	.	.	.	.	.	.	.	.	.	.	.	.	.	M	.	.	.	.	.	.	.	.	.	.	.				
chicken/Kudus/018/10	.	.	.	.	.	.	.	.	.	.	.	V	.	.	.	.	.	.	.	.	.	.	.	.	.	.	.	.	M	.	.	.	.	.	.	.	.	.	.	.				
chicken/Sweden/97	.	.	.	.	.	.	.	.	.	.	.	.	.	.	.	.	.	.	.	.	.	.	.	.	.	.	.	.	M	.	.	.	.	.	.	.	.	.	.	.				
Sterna/Astr/2755/2001	.	.	.	.	.	.	.	.	.	.	.	.	.	.	.	.	.	.	.	.	.	.	.	.	.	.	.	.	M	.	.	.	.	.	.	.	.	.	.	.				
TW-99-175	.	.	.	.	.	.	.	.	.	.	.	.	.	.	.	.	.	.	.	.	.	.	.	.	.	.	.	.	M	.	.	.	.	.	.	.	.	.	.	.				
XJ-2/97	.	.	.	.	.	.	.	.	.	.	.	V	.	.	.	.	.	.	.	.	.	.	.	.	.	.	.	.	M	.	R	.	.	.	.	.	.	.	.	.				
JS/1/97/Go	.	.	.	.	.	.	.	.	.	.	.	.	.	.	.	.	.	.	.	.	.	.	.	.	.	.	.	.	M	.	.	.	.	.	.	.	.	.	.	.				
BP01	.	.	.	.	.	.	.	.	.	.	.	V	.	.	.	.	.	.	.	.	.	.	.	.	.	.	.	.	M	.	R	.	.	.	.	.	.	.	.	.				
JS/5/06/Go	.	.	.	.	.	.	.	.	.	.	.	V	.	.	.	.	.	.	.	.	.	.	.	.	.	.	.	.	M	.	R	.	.	.	.	.	.	.	.	.				
HA-14-07-Ch	.	.	.	.	.	.	.	.	.	.	.	V	.	.	.	.	.	.	.	.	.	.	.	.	.	.	.	.	M	.	R	.	.	.	.	.	.	.	.	.				
JSD0812	.	.	.	.	.	.	.	.	.	.	.	V	.	.	.	.	.	.	.	.	.	.	.	.	.	.	.	.	M	.	R	.	.	.	.	.	.	.	.	.				
sh09	.	.	.	.	.	.	.	.	.	.	.	V	.	.	.	.	.	.	.	.	.	.	.	.	.	.	.	.	M	.	R	.	.	.	.	.	.	.	.	.				
chicken/China/JSX1/2010	.	.	.	.	.	.	.	.	.	.	.	V	.	.	.	.	.	.	.	.	.	.	.	.	.	.	.	.	M	.	R	.	.	.	.	.	.	.	.	.				
JS-12-11-Ch	.	.	.	.	.	.	.	.	.	.	.	V	.	.	.	.	.	.	.	.	.	.	.	.	.	.	.	.	M	.	R	.	.	.	.	.	.	.	.	.				
NDV/Chicken/TC/1/2011	.	.	.	.	.	.	.	.	.	.	S	V	.	.	.	.	.	.	.	.	.	.	.	.	.	.	.	.	M	.	R	.	.	.	.	.	.	.	.	.				
NDV/Chicken/TC/2/2011	.	.	.	.	.	.	.	.	.	.	S	V	.	.	.	.	.	.	.	.	.	.	.	.	.	.	.	.	M	.	R	.	.	.	.	.	.	.	.	.				
NDV/Chicken/TC/3/2011	.	.	.	.	.	.	.	.	.	.	S	V	.	.	.	.	.	.	.	.	.	.	.	.	.	.	.	.	M	.	R	.	.	.	.	.	.	.	.	.				
NDV/Chicken/TC/4/2011	.	.	.	.	.	.	.	.	.	.	S	V	.	.	.	.	.	.	.	.	.	.	.	.	.	.	.	.	M	.	R	.	.	.	.	.	.	.	.	.				
NDV/Chicken/TC/5/2011	.	.	.	.	.	.	.	.	.	.	S	V	.	.	.	.	.	.	.	.	.	.	.	.	.	.	.	.	M	.	R	.	.	.	.	.	.	.	.	.				
NDV/Chicken/TC/6/2011	.	.	.	.	.	.	.	.	.	.	S	V	.	.	.	.	.	.	.	.	.	.	.	.	.	.	.	.	M	.	R	.	.	.	.	.	.	.	.	.				
NDV/Chicken/TC/7/2011	.	.	.	.	.	.	.	.	.	.	S	V	.	.	.	.	.	.	.	.	.	.	.	.	.	.	.	.	M	.	R	.	.	.	.	.	.	.	.	.				
NDV/Chicken/TC/8/2011	.	.	.	.	.	.	.	.	.	.	S	V	.	.	.	.	.	.	.	.	.	.	.	.	.	.	.	.	M	.	R	.	.	.	.	.	.	.	.	.				
NDV/Chicken/TC/9/2011	.	.	.	.	.	.	.	.	.	.	S	V	.	.	.	.	.	.	.	.	.	.	.	.	.	.	.	.	M	.	R	.	.	.	.	.	.	.	.	.				
PX2/03	.	.	.	.	.	.	.	.	.	.	.	V	.	.	.	.	.	.	.	.	.	.	.	.	.	.	.	.	M	.	R	.	.	.	.	.	.	.	.	.	.			
Chicken/China/Hebei/02/2012	.	.	.	.	.	.	.	.	.	.	.	V	.	.	.	.	.	.	.	.	.	.	.	.	.	.	.	.	M	.	R	.	.	.	.	.	.	.	.	.	.			
Shangdong/02/2012	.	.	.	.	.	.	.	.	.	.	.	V	.	.	.	.	.	.	.	.	.	.	.	.	.	.	.	.	M	.	R	.	.	.	.	.	.	R	.	.	.			
YZCQ/Liaoning/08	.	.	.	.	.	.	.	.	.	.	.	V	.	.	.	.	.	.	.	.	.	.	.	.	.	.	.	.	M	.	.	.	.	.	.	.	.	.	D	.	.			
Beijing/01/2012	.	.	.	.	.	.	.	.	.	.	.	V	.	.	.	.	.	.	.	.	.	.	.	.	.	.	.	.	M	.	R	.	.	.	.	.	.	R	.	.	.			
SD-02-11-Ch	.	.	.	.	.	.	.	.	.	.	.	V	.	.	.	.	.	.	.	.	.	.	.	.	.	.	.	.	M	.	R	.	.	.	.	.	.	R	.	.	.			
Ch/SD672/12	.	.	.	.	.	.	.	.	.	.	.	V	.	.	.	.	.	.	.	.	.	.	.	.	.	.	.	.	M	.	R	.	.	.	.	.	.	.	.	.				
Ch/SD754/12	.	.	.	.	.	.	.	.	.	.	.	V	.	.	.	.	.	.	.	.	.	.	.	.	.	.	.	.	M	.	R	.	.	.	.	.	.	R	.	.	.			
Ch/SD755/12	.	.	.	.	.	.	.	.	.	.	.	V	.	.	.	.	.	.	.	.	.	.	.	.	.	.	.	.	M	.	R	.	.	.	.	.	.	R	.	.	.			
Ch/SD758/12	.	.	.	.	.	.	.	.	.	.	.	V	.	.	.	.	.	.	.	.	.	.	.	.	.	.	.	.	M	.	R	.	.	.	.	.	.	R	.	.	.			
Ch/SD01/13	.	.	.	.	.	.	.	.	.	.	.	V	.	.	L	.	.	.	.	.	.	.	.	.	.	.	.	.	M	.	R	.	.	.	.	.	.	R	.	.	.			
TW/99-156B	.	.	.	.	.	.	.	.	.	.	.	V	.	.	.	.	.	.	.	.	.	.	.	.	.	.	.	.	I	.	.	.	.	.	.	.	.	.	.	.				
TW-02-301	.	.	.	.	.	.	.	.	.	.	S	V	.	.	.	.	.	.	.	.	.	.	.	.	.	.	.	.	M	.	.	.	.	.	.	.	.	.	.	.				
TW-02-302	.	.	.	.	.	.	.	.	.	.	S	V	.	.	.	.	.	.	.	.	.	.	.	.	.	.	.	.	M	.	.	.	.	.	.	.	.	.	.	.				
TW-04-CB8	.	.	.	.	.	.	.	.	.	.	S	V	.	.	.	.	.	.	.	.	.	.	.	.	.	.	.	.	M	.	.	.	.	G	.	.	.	.	.	.				
TW/08-02	.	.	.	.	.	.	.	.	.	.	S	V	.	.	.	.	.	.	.	.	.	.	.	.	.	.	.	.	M	.	.	.	.	.	G	.	.	.	.	.				
strains	Amino acid residues																																											
																				101										112				117		121								
Clone 30	L	D	A	Y	N	R	T	L	T	T	L	L	T	P	L	G	D	S	I	R	R	I	Q	E	S	V	T	T	S	G	G	G	R	Q	G	R	L	I	G	A	I	I	G	123
B1	.	.	.	.	.	.	.	.	.	.	.	.	.	.	.	.	.	.	.	.	.	.	.	.	.	.	.	.	.	.	.	.	.	.	.	.	.	.	.	.	.	.	.	
LaSota	.	.	.	.	.	.	.	.	.	.	.	.	.	.	.	.	.	.	.	.	.	.	.	.	.	.	.	.	.	.	.	.	.	.	.	.	.	.	.	.	.	.	.	
Mukteswar	.	E	.	.	.	.	.	.	.	.	.	.	.	.	.	.	.	.	.	.	.	.	.	.	.	.	.	.	.	.	.	R	.	.	R	.	F	.	.	.	.	.	.	
Herts/33	.	E	.	.	.	.	.	.	.	.	.	.	.	.	.	.	.	.	.	.	.	.	.	.	.	.	.	.	.	.	.	R	.	.	R	.	F	.	.	.	.	.	.	
NL/152608/93	.	E	.	.	.	.	.	.	.	.	.	.	.	.	.	.	.	.	.	.	K	.	.	G	.	.	S	.	P	.	.	R	.	.	K	.	F	.	.	.	V	.	.	
chicken/Kudus/018/10	.	E	.	.	.	.	.	.	.	.	.	.	.	.	.	.	.	.	.	.	K	.	.	G	.	.	A	.	.	.	.	R	.	.	K	.	F	.	.	.	V	.	.	
chicken/Sweden/97	.	E	.	.	.	.	.	.	.	.	.	.	.	.	.	.	.	.	.	.	.	.	.	G	.	.	S	.	.	.	.	R	.	.	R	.	F	.	.	.	V	.	.	
Sterna/Astr/2755/2001	.	E	.	.	.	.	.	.	.	.	.	.	.	.	.	.	.	.	.	.	.	.	.	G	.	.	S	.	.	.	.	R	.	.	R	.	F	.	.	.	V	.	.	
TW-99-175	.	E	.	.	.	.	.	.	.	.	.	.	.	.	.	.	.	.	.	.	K	.	.	G	.	.	S	.	.	.	.	R	.	.	K	.	F	.	.	.	V	.	.	
XJ-2/97	.	E	.	.	.	.	.	.	.	.	.	.	.	.	.	.	.	.	.	.	K	.	.	G	.	.	S	.	.	.	.	R	.	.	K	.	F	.	.	.	V	.	.	
JS/1/97/Go	.	E	.	.	.	.	.	.	.	.	.	.	.	.	.	.	.	.	.	.	.	.	.	.	.	A	.	.	.	.	.	R	.	.	R	.	F	.	.	.	.	.	.	
BP01	.	E	.	.	.	.	.	.	.	A	.	.	.	.	.	.	.	.	.	.	K	.	.	G	.	.	S	.	.	.	.	R	.	.	K	.	F	.	.	.	V	.	.	
JS/5/06/Go	.	E	.	.	.	.	.	.	.	.	.	.	.	.	.	.	.	.	.	.	K	.	.	G	.	.	S	.	.	.	.	R	.	.	K	.	F	.	.	.	V	.	.	
HA-14-07-Ch	.	E	.	.	.	.	.	.	.	.	.	.	.	.	.	.	.	.	.	.	K	.	.	G	.	.	S	.	.	.	.	R	.	.	K	.	F	.	.	.	V	.	.	
JSD0812	.	E	.	.	.	.	.	.	.	.	.	.	.	.	.	.	.	.	.	.	K	.	.	G	.	.	S	.	.	.	.	R	.	.	K	.	F	.	.	.	V	.	.	
sh09	.	E	.	.	.	.	.	.	.	.	.	.	.	.	.	.	.	.	.	.	K	.	.	G	.	.	Y	.	.	.	.	R	.	.	K	.	F	.	.	.	V	.	.	
chicken/China/JSX1/2010	.	E	.	.	.	.	.	.	.	A	.	.	.	.	.	.	.	.	.	.	K	.	.	G	.	.	S	.	.	.	.	R	.	.	K	.	F	.	.	.	V	.	.	
JS-12-11-Ch	.	E	.	.	.	.	.	.	.	.	.	.	.	.	.	.	.	.	.	.	K	.	.	G	.	.	S	.	.	.	.	R	.	.	K	.	F	.	.	.	V	.	.	
NDV/Chicken/TC/1/2011	.	E	.	.	.	.	.	.	.	.	.	.	.	.	.	.	.	.	.	.	K	.	.	G	.	.	S	.	.	.	.	R	.	.	K	.	F	.	.	.	V	.	.	
NDV/Chicken/TC/2/2011	.	E	.	.	.	.	.	.	.	.	.	.	.	.	.	.	.	.	.	.	K	.	.	G	.	.	S	.	.	.	.	R	.	.	K	.	F	.	.	.	V	.	.	
NDV/Chicken/TC/3/2011	.	E	.	.	.	.	.	.	.	.	.	.	.	.	.	.	.	.	.	.	K	.	.	G	.	.	S	.	.	.	.	R	.	.	K	.	F	.	.	.	V	.	.	
NDV/Chicken/TC/4/2011	.	E	.	.	.	.	.	.	.	.	.	.	.	.	.	.	.	.	.	.	K	.	.	G	.	.	S	.	.	.	.	R	.	.	K	.	F	.	.	.	V	.	.	
NDV/Chicken/TC/5/2011	.	E	.	.	.	.	.	.	.	.	.	.	.	.	.	.	.	.	.	.	K	.	.	G	.	.	S	.	.	.	.	R	.	.	K	.	F	.	.	.	V	.	.	
NDV/Chicken/TC/6/2011	.	E	.	.	.	.	.	.	.	.	.	.	.	.	.	.	.	.	.	.	K	.	.	G	.	.	S	.	.	.	.	R	.	.	K	.	F	.	.	.	V	.	.	
NDV/Chicken/TC/7/2011	.	E	.	.	.	.	.	.	.	.	.	.	.	.	.	.	.	.	.	.	K	.	.	G	.	.	S	.	.	.	.	R	.	.	K	.	F	.	.	.	V	.	.	
KNDV/Chicken/TC/8/2011	.	E	.	.	.	.	.	.	.	.	.	.	.	.	.	.	.	.	.	.	K	.	.	G	.	.	S	.	.	.	.	R	.	.	K	.	F	.	.	.	V	.	.	
NDV/Chicken/TC/9/2011	.	E	.	.	.	.	.	.	.	.	.	.	.	.	.	.	.	.	.	.	K	.	.	G	.	.	S	.	.	.	.	R	.	.	K	.	F	.	.	.	V	.	.	
PX2/03	.	E	.	.	.	.	.	.	.	.	.	.	.	.	.	.	.	.	.	.	K	.	.	G	.	.	S	.	.	G	.	R	.	.	K	.	F	.	.	.	V	.	.	
Hebei/02/2012	.	E	.	.	.	.	.	.	.	.	.	.	.	.	.	.	.	.	.	.	K	.	.	G	.	.	S	.	.	.	.	R	.	.	K	.	F	.	.	.	V	.	.	
Shangdong/02/2012	.	E	.	.	.	.	.	.	.	.	.	.	.	.	.	.	.	.	.	.	K	.	.	G	.	.	S	.	.	.	.	R	.	.	K	.	F	.	.	.	V	.	.	
YZCQ/Liaoning/08	.	E	.	.	.	.	.	.	.	.	.	.	.	.	.	.	E	.	.	.	K	.	.	G	.	.	S	.	.	.	.	R	.	.	K	.	F	.	.	.	V	.	.	
Beijing/01/2012	.	E	.	.	.	.	.	.	.	.	.	.	.	.	.	.	.	.	.	.	K	.	.	G	.	.	S	.	.	.	.	R	.	.	K	.	F	.	.	.	V	.	.	
SD-02-11-Ch	.	E	.	.	.	.	.	.	.	.	.	.	.	.	.	.	.	.	.	.	K	.	.	G	.	.	S	.	.	.	.	R	.	.	K	.	F	.	.	.	V	.	.	
Ch/SD672/12	.	E	.	.	.	.	.	.	.	.	.	.	.	.	.	.	.	.	.	.	K	.	.	G	.	.	S	.	.	.	.	R	.	.	K	.	F	.	.	.	V	.	.	
Ch/SD754/12	.	E	.	.	.	.	.	.	.	.	.	.	.	.	.	.	.	.	.	.	K	.	.	G	.	.	S	.	.	.	.	R	.	.	K	.	F	.	.		V	.	.	
Ch/SD755/12	.	E	.	.	.	.	.	.	.	.	.	.	.	.	.	.	.	.	.	.	K	.	.	G	.	.	S	.	.	.	.	R	.	.	K	.	F	.	.	.	V	.	.	
Ch/SD758/12	.	E	.	.	.	.	.	.	.	.	.	.	.	.	.	.	.	.	.	.	K	.	.	G	.	.	S	.	.	.	.	R	.	.	K	.	F	.	.	.	V	.	.	
Ch/SD01/13	.	E	.	.	.	.	.	.	.	.	.	.	.	.	.	.	.	.	.	.	K	.	.	G	.	.	S	.	.	.	.	R	.	.	K	.	F	.	.	.	V	.	.	
TW/99-156B	.	E	.	.	.	.	.	.	.	.	.	.	.	.	.	.	.	.	.	.	K	.	.	G	.	.	S	.	.	.	.	R	.	.	K	.	F	.	.	.	V	.	.	
TW-02-301	.	E	.	.	.	.	.	.	.	A	.	.	.	.	.	.	.	.	.	.	K	.	.	G	.	.	S	.	.	.	.	R	.	.	K	.	F	.	.	.	V	.	.	
TW-02-302	.	E	.	.	.	.	.	.	.	A	.	.	.	.	.	.	.	.	.	.	K	.	.	G	.	.	S	.	.	.	.	R	.	.	K	.	F	.	.	.	V	.	.	
TW-04-CB8	.	E	.	.	.	.	.	.	.	.	.	.	.	.	.	.	.	.	.	.	K	.	.	G	.	.	S	.	.	.	.	R	.	K	K	.	F	.	.	.	V	.	.	
TW/08-02	.	E	.	.	.	.	.	.	.	A	.	.	.	.	.	.	.	.	.	.	K	.	.	G	.	.	S	.	.	.	.	R	.	.	K	.	F	.	.	.	V	.	.	

Only sequences different from the NDV strain Clone 30 are shown. The sequences at the fusion cleavage site and residues discussed in the text are underlined

Residues that are identical to the NDV strain Clone 30 are indicated by a dot (.). The other published sequences were indicated in Table 1 and described previously [2, 5]. NL/152608/93: APMV-1/chicken/NL/152608/93; Hebei/01/2012: chicken/China/ Hebei/01/2012; Shandong/02/2012: chicken/China/ Shandong/02/2012; Beijing/01/2012: chicken/China/ Beijing/ 01/2012

The nucleotide and predicted amino acid sequences of the F gene of NDVs were aligned. The aligned sequences were then used in pairwise comparisons and the percentage of nucleotide and amino acid sequence identities were determined.

The percentage of nucleotide sequence identity of F gene among these Shaanxi strains was in the range of 98.9 %–99.6 %, while the percentage of amino acid sequence identity was in the range of 98.4 %–99.5 %. Sequence comparisons of Shaanxi strains with five vaccine strains (Herts/33, B1, Clone 30, LaSota, and Mukteswar) homologues revealed that there exist extensive sequence divergence, with 80.7 %–87.2 % and 81.9 %–88.1 % identities of nucleotide and amino acid sequence, respectively. The Shaanxi strains exhibited higher sequence identity with Herts/33 vaccine strain. The percentage of nucleotide sequence identity was in the range of 86.6 %–87.2 %, while the percentage of amino acid sequence identity was in the range of 87.6 %–88.1 %. Furthermore, Shaanxi strains showed the lowest sequence identity with B1 vaccine strain, with 80.7 %–81.3 % and 81.9 %–82.4 % identity of nucleotide and amino acid sequence, respectively. Additionally, the percentage of nucleotide sequence identity of the F gene of NDV between Shaanxi and LaSota vaccine strains was in the range of 81.7 %–82.3 %, while the percentage of amino acid sequence identity of NDV F protein was in the range of 82.8 %–83.3 %.

Comparison of the deduced amino acid sequences of the F protein of recent Shaanxi strains with the traditional vaccine strains revealed that there were many amino acid residue substitutions at F protein (Table 2). Interestingly, compared to the past pandemic strains in China during the past 10 years [16–20], a number of amino acid residue substitutions at N-terminal of F protein were found in Shaanxi NDV strains. Examples include amino acid residue substitutions of all NDV at positions 13 (M to L and M to P), 14 (M to V), 19 (I to V or T to V), 22 (V to A, V to T and V to I), 28 (P to L, P to T and P to S), and 51 (N to S) (Table 2). It is worth to note that two of amino acid residues at positions 14(V) and 51(S) were identified as specific for recent Shaanxi NDV strains in compared to the past pandemic strains in China (Table 2). Taken together, our results of the present work provide evidences suggesting that extensive sequence divergence occur among Shaanxi strains, past pandemic strains in China, and vaccine strains.

### Sequence analysis of NDV isolates of HN gene of NDV

Based on the C-terminal HN extension of NDV, 4 groups were classified, including amino acid residues of 571, 577, 580, and 616 [14]. In the current study, we found that recent Shaanxi strains have HN gene encoding only 571 amino acid residues of HN glycoprotein. The percentage of nucleotide sequence identity of HN gene among recent Shaanxi strains was in the range of 99.0 %–99.7 %, while the percentage of amino acid sequence identity was in the range of 98.5 %–99.6 %. Furthermore, the percentage of nucleotide sequence identity of HN gene between Shaanxi and vaccine strains was in the range of 81.4 %–84.8 %, while the percentage of amino acid sequence identity was in the range of 87.5 %–91.2 %. Sequence analysis of Shaanxi and vaccine strains exhibited that recent Shaanxi strains of NDV had the lowest percentage of sequence identity with LaSota vaccine strain. The percentage of nucleotide and amino acid sequence identity between recent Shaanxi and LaSota vaccine strains was in the range of 81.4 %–81.6 % and 87.5 %–88.0 %, respectively.

### Amino acid residue substitutions at neutralizing epitopes on the HN glycoprotein among Shannxi isolates of NDV

Analysis of antigenic sites on the HN glycoprotein of recent Shaanxi NDV strains revealed that there are many amino acid residue substitutions at neutralizing epitopes on HN protein (Table 3). As compared to vaccine strains (Herts/33, B1, Clone 30, LaSota, and Mukteswar), many amino acid residue substitutions at positions 347E → R (sites 1 and 14), 350Y → H (sites 1 and 14), 494G → D (site 1 2), 514I → V (sites 2 and 12), 518S → N (sites 2 and 12), 519S → L (sites 2 and 12) were found in recent Shaanxi strains of NDV (Table 3). Furthermore, in comparison to the past pandemic strains in China during the past 10 years, several amino acid residue substitutions at neutralizing epitopes on HN protein were uncovered in recent Shaanxi strains. Examples include amino acid residue substitutions at positions 347 (E to R) with JSD0812 whose host was duck and 347 (E to R) with sh09 whose host were chickens as well as 518S → N (sites 2 and 12) and 519S → L (sites 2 and 12) (Table 3). Additionally, compared to the recent Taiwanese NDV isolates, several amino acid residue substitutions at neutralizing epitopes on the HN protein at positions 347G → R, 348H → Q, 350Y → H, 352 T → I, 518S → N and 519S → L were also uncovered (Table 3). It is interesting to note that 4 amino acid residues at positions 347(R), 350(H), 518 (N), and 519 (L) were conserved in recent Shaanxi NDV isolates and identified as a specific marker for these isolates (Table 3).

**Table 3:** Alignment of HN amino acid sequences of recent Taiwanese isolates and previously published virus strains was performed using the DNASTAR software

	Amino acid residues																															
Strains	HR-A 74-89												HR-B 96-110																			
Clone 30	L	G	S	N	Q	D	V	V	D	R	I	Y	K	Q	V	A	L	L	N	T	E	T	T	I	M	N	A	I		S	L	
B1	.	.	.	.	.	.	.	.	.	.	.	.	.	.	.	.	.	.	.	.	.	.	.	.	.	.	.	.	.	.	.	
LaSota	.	.	.	.	.	.	.	.	.	.	.	.	.	.	.	.	.	.	K	.	.	.	.	.	.	.	.	.	.	.	.	
Mukteswar	.	.	.	.	.	.	.	.	.	.	.	.	.	.	.	.	.	.	.	.	.	S	I	.	.	.	.	.	.	.	.	
Herts/33	.	S	.	.	.	.	.	.	.	.	.	.	.	.	.	.	.	.	.	.	.	S	V	.	.	.	.	.	.	.	.	
NL/152608/93	.	S	.	S	.	.	.	I	.	.	.	.	.	.	.	.	.	.	.	.	.	S	I	.	.	.	.	.	.	.	.	
chicken/Kudus/018/10	.	S	.	S	.	.	.	I	.	.	.	.	.	.	.	.	.	.	.	.	.	S	M	.	.	.	.	.	.	.	.	
chicken/Sweden/97	.	S	.	S	.	.	.	I	.	.	.	.	.	.	.	.	.	.	.	.	.	S	I	.	.	.	.	.	.	.	.	
Sterna/Astr/2755/2001	.	.	.	S	.	.	.	I	.	.	.	.	.	.	.	.	.	.	.	.	.	S	I	.	.	.	.	.	.	.	.	
TW-99-175	.	S	.	S	.	.	.	I	.	.	.	.	.	.	.	.	.	.	.	.	.	S	I	.	.	.	.	.	.	.	.	
XJ-2/97	.	S	.	S	.	.	.	I	.	.	.	.	.	.	.	.	.	.	.	.	.	S	I	.	.	.	.	.	.	.	.	
JS/1/97/Go	.	S	.	S	.	.	.	I	.	.	.	.	.	.	.	.	.	.	.	.	.	S	I	.	.	.	.	.	.	.	.	
BP01	.	S	.	S	.	.	.	I	.	.	.	.	.	.	.	.	.	.	.	.	.	S	I	.	.	.	.	.	.	.	.	
JS/5/06/Go	.	S	.	S	.	.	.	I	.	.	.	.	.	.	.	.	.	.	.	.	.	S	.	.	.	.	.	.	.	.	.	
HA-14-07-Ch	.	S	.	S	.	.	.	I	.	.	.	.	.	.	.	.	.	.	.	.	.	S	.	.	.	.	.	.	.	.	.	
JSD0812	.	S	.	S	.	.	.	I	.	.	.	.	.	.	.	.	.	.	.	.	.	S	I	.	.	.	.	.	.	.	.	
sh09	.	S	.	S	.	.	.	I	.	.	.	.	.	.	.	.	.	.	.	.	.	S	I	.	.	.	.	.	.	.	.	
chicken/China/JSX1/2010	.	S	.	S	.	.	.	I	.	.	.	.	.	.	.	.	.	.	.	.	.	S	.	.	.	.	.	.	.	.	.	
JS-12-11-Ch	.	S	.	S	.	.	.	I	.	.	.	.	.	.	.	.	.	.	.	.	.	S	I	.	.	.	.	.	.	.	.	
NDV/Chicken/TC/1/2011	.	S	.	S	.	.	.	I	.	.	.	.	.	.	.	.	.	.	.	.	.	S	.	.	.	.	.	.	.	.	.	
NDV/Chicken/TC/2/2011	.	S	.	S	.	.	.	I	.	.	.	.	.	.	.	.	.	.	.	.	.	S	.	.	.	.	.	.	.	.	.	
NDV/Chicken/TC/3/2011	.	S	.	S	.	.	.	I	.	.	.	.	.	.	.	.	.	.	.	.	.	S	.	.	.	.	.	.	.	.	.	
NDV/Chicken/TC/4/2011	.	S	.	S	.	.	.	I	.	.	.	.	.	.	.	.	.	.	.	.	.	S	.	.	.	.	.	.	.	.	.	
NDV/Chicken/TC/5/2011	.	S	.	S	.	.	.	I	.	.	.	.	.	.	.	.	.	.	.	.	.	S	.	.	.	.	.	.	.	.	.	
NDV/Chicken/TC/6/2011	.	S	.	S	.	.	.	I	.	.	.	.	.	.	.	.	.	.	.	.	.	S	.	.	.	.	.	.	.	.	.	
NDV/Chicken/TC/7/2011	.	S	.	S	.	.	.	I	.	.	.	.	.	.	.	.	.	.	.	.	.	S	.	.	.	.	.	.	.	.	.	
NDV/Chicken/TC/8/2011	.	S	.	S	.	.	.	I	.	.	.	.	.	.	.	.	.	.	.	.	.	S	.	.	.	.	.	.	.	.	.	
NDV/Chicken/TC/9/2011	.	S	.	S	.	.	.	I	.	.	.	.	.	.	.	.	.	.	.	.	.	S	.	.	.	.	.	.	.	.	.	
PX2/03	.	S	.	S	.	.	.	I	.	.	.	.	.	.	.	.	.	.	.	.	.	S	I	.	.	.	.	.	.	.	.	
Chicken/China/Hebei/02/2012	.	S	.	S	.	.	.	I	.	.	.	.	.	.	.	.	.	.	.	.	.	S	I	.	.	.	.	.	.	.	.	
Shangdong/02/2012	.	S	.	G	.	.	.	I	.	.	.	.	.	.	.	.	.	.	.	.	.	S	V	.	.	.	.	.	.	.	.	
YZCQ/Liaoning/08	.	S	.	S	.	.	.	I	.	.	.	.	.	.	.	.	.	.	.	.	.	S	M	.	.	.	.	.	.	.	.	
Beijing/01/2012	.	S	.	S	.	.	.	I	.	.	.	.	.	.	.	.	.	.	.	.	.	S	V	.	.	.	.	.	.	.	.	
SD-02-11-Ch	.	S	.	G	.	.	.	I	.	.	.	.	.	.	.	.	.	.	.	.	.	S	V	.	.	.	.	.	.	.	.	
Ch/SD672/12	.	S	.	G	.	.	.	I	.	.	.	.	.	.	.	.	.	.	.	.	.	S	V	.	.	.	.	.	.	.	.	
Ch/SD754/12	.	S	.	G	.	.	.	I	.	.	.	.	.	.	.	.	.	.	.	.	.	S	V	.	.	.	.	.	.	.	.	
Ch/SD755/12	.	S	.	G	.	.	.	I	.	.	.	.	.	.	.	.	.	.	.	.	.	S	V	.	.	.	.	.	.	.	.	
Ch/SD758/12	.	S	.	S	.	.	.	I	.	.	.	.	.	.	.	.	.	.	.	.	.	S	V	.	.	.	.	.	.	.	.	
Ch/SD01/13	.	S	.	G	.	.	.	I	.	.	.	.	.	.	.	.	.	.	.	.	.	S	V	.	.	.	.	.	.	.	.	
TW/99-156B	-	-	-	-	-	-	-	-	-	-	-	-	-	-	-	-	-	-	-	-	-	-	-	-	-	-	-	-	-	-	-	
TW-02-301	.	S	.	S	.	.	.	M	.	.	.	.	.	.	.	.	.	.	.	.	.	S	I	.	.	.	.	.	.	.	.	
TW-02-302	.	S	.	S	.	.	.	M	.	.	.	.	.	.	.	.	.	.	.	.	.	S	I	.	.	.	.	.	.	.	.	
TW-04-CB8	.	S	.	S	.	.	.	M	.	.	.	.	.	.	.	.	.	.	.	.	.	S	I	.	.	.	.	.	.	.	.	
TW/08-02	.	S	.	S	.	.	.	M	.	.	.	.	.	.	.	.	.	.	.	.	.	S	I	.	.	.	.	.	.	.	.	
	Amino acid residues																															
Strains	Site 23							Site 1 and 14					Site 12		Site 2 and 12						Site 2											
Clone 30	193-201							345-355					494		513-521						569											
	L	S	G	C	R	D	H	S	H	P	D	E	Q	D	Y	Q	I	R	M	A		G	R	I	T	R	V	S		S	S	D
B1	.	.	.	.	.	.	.	.	.	.	.	.	.	.	.	.	.	.	.	.		.	.	.	.	.	.	.	.	.	.	.
LaSota	.	.	.	.	.	.	.	.	.	.	.	.	.	.	.	.	.	.	.	.		.	.	.	.	.	.	.	.	.	.	.
Mukteswar	.	.	.	.	.	.	.	.	.	.	.	.	.	.	.	.	.	.	.	.		D	.	.	.	.	.	.	.	.	.	G
Herts/33	.	.	.	.	.	.	.	.	.	.	.	.	.	.	.	.	.	.	.	.		D	.	.	.	.	.	.	.	R	.	.
NL/152608/93	.	.	.	.	.	.	.	.	.	.	.	.	.	.	.	.	.	.	.	.		D	.	V	.	.	.	.	.	.	.	.
chicken/Kudus/018/10	.	.	.	.	.	.	.	.	.	.	.	.	.	.	.	.	.	.	.	.		D	.	V	.	.	.	.	.	.	.	N
chicken/Sweden/97	.	.	.	.	.	.	.	.	.	.	.	.	.	.	.	.	.	.	.	.		D	.	V	.	Q	.	.	.	.	.	A
Sterna/Astr/2755/2001	.	.	.	.	.	.	.	.	.	.	.	.	.	.	.	.	.	.	.	.		D	.	V	.	.	.	.	.	.	.	V
TW-99-175	.	.	.	.	.	.	.	.	.	.	.	G	.	.	.	.	.	.	.	.		D	.	V	.	.	.	.	.	.	.	.
XJ-2/97	.	.	.	.	.	.	.	.	.	.	.	.	.	.	.	.	.	.	.	.		D	.	V	.	.	.	.	.	.	.	.
JS/1/97/Go	.	.	.	.	.	.	.	.	.	.	.	G	.	.	.	.	.	.	.	.		D	.	V	.	.	.	.	.	.	.	.
BP01	.	.	.	.	.	.	.	.	.	.	.	.	.	.	.	.	.	.	.	.		D	.	V	.	.	.	.	.	.	.	.
JS/5/06/Go	.	.	.	.	.	.	.	.	.	.	.	G	.	.	.	.	.	.	.	.		D	.	V	.	.	.	.	.	.	.	.
HA-14-07-Ch	.	.	D	.	.	.	.	.	.	.	.	G	.	.	.	.	.	.	.	.		D	.	V	.	.	.	.	.	.	.	.
JSD0812	.	.	.	.	.	.	.	.	.	.	.	.	.	.	.	.	.	.	.	.		D	.	V	.	.	.	.	.	.	.	.
sh09	.	.	.	.	.	.	.	.	.	.	.	.	.	.	.	.	.	.	.	.		D	.	V	.	.	.	.	.	.	.	.
chicken/China/JSX1/2010	.	.	.	.	.	.	.	.	.	.	.	G	.	.	H	.	.	.	.	.		D	.	V	.	.	.	.	.	.	.	.
JS-12-11-Ch	.	.	.	.	.	.	.	.	.	.	.	.	.	.	.	.	.	.	.	.		D	.	V	.	.	.	.	.	.	.	.
NDV/Chicken/TC/1/2011	.	.	.	.	.	.	.	.	.	.	.	R	.	.	H	.	.	.	.	.		D	.	V	.	.	.	N	L	.	.	.
NDV/Chicken/TC/2/2011	.	.	.	.	.	.	.	.	.	.	.	R	.	.	H	.	.	.	.	.		D	.	V	.	.	.	N	L	.	.	.
NDV/Chicken/TC/3/2011	.	.	.	.	.	.	.	.	.	.	.	R	.	.	H	.	.	.	.	.		D	.	V	.	.	.	N	L	.	.	.
NDV/Chicken/TC/4/2011	.	.	.	.	.	.	.	.	.	.	.	R	.	.	H	.	.	.	.	.		D	.	V	.	.	.	N	L	.	.	.
NDV/Chicken/TC/5/2011	.	.	.	.	.	.	.	.	.	.	.	R	.	.	H	.	.	.	.	.		D	.	V	.	.	.	N	L	.	.	.
NDV/Chicken/TC/6/2011	.	.	.	.	.	.	.	.	.	.	.	R	.	.	H	.	.	.	.	.		D	.	V	.	.	.	N	L	.	.	.
NDV/Chicken/TC/7/2011	.	.	.	.	.	.	.	.	.	.	.	R	.	.	H	.	.	.	.	.		D	.	V	.	.	.	N	L	.	.	.
NDV/Chicken/TC/8/2011	.	.	.	.	.	.	.	.	.	.	.	R	.	.	H	.	.	.	.	.		D	.	V	.	.	.	N	L	.	.	.
NDV/Chicken/TC/9/2011	.	.	.	.	.	.	.	.	.	.	.	R	.	.	H	.	.	.	.	.		D	.	V	.	.	.	N	L	.	.	.
PX2/03	.	.	.	.	.	.	.	.	.	.	.	.	.	.	.	.	.	.	.	.		D	.	V	.	.	.	.	.	.	.	.
Chicken/China/Hebei/02/2012	.	.	.	.	.	.	.	.	.	.	.	.	.	.	.	.	.	.	.	.		D	.	V	.	.	.	.	.	.	.	.
Shangdong/02/2012	.	.	.	.	.	.	.	.	.	.	.	K	.	.	.	.	.	.	.	.		D	.	V	.	.	.	.	.	.	.	.
YZCQ/Liaoning/08	.	.	.	.	.	.	.	.	.	.	.	K	.	.	.	.	.	.	.	.		D	.	V	.	.	.	.	.	.	.	.
Beijing/01/2012	.	.	.	.	.	.	.	.	.	.	.	K	.	.	.	.	.	.	.	.		D	.	V	.	.	.	.	.	.	.	.
SD-02-11-Ch	.	.	.	.	G	.	.	.	.	.	.	K	.	.	.	.	.	.	.	.		D	.	V	.	.	.	.	.	.	.	.
Ch/SD672/12	.	.	.	.	.	.	.	.	.	.	.	K	.	.	.	.	.	.	.	.		D	.	V	.	.	.	.	.	.	.	.
Ch/SD754/12	.	.	.	.	.	.	.	.	.	.	.	K	.	.	.	.	.	.	.	.		D	.	V	.	.	.	.	.	.	.	.
Ch/SD755/12	.	.	.	.	.	.	.	.	.	.	.	K	.	.	.	.	.	.	.	.		D	.	V	.	.	.	.	.	.	.	.
Ch/SD758/12	.	.	.	.	.	.	.	.	.	.	.	K	.	.	.	.	.	.	.	.		D	.	V	.	.	.	.	.	.	.	.
Ch/SD01/13	.	.	.	.	.	.	.	.	.	.	.	K	.	.	.	.	.	.	.	.		D	.	V	.	.	.	.	.	.	.	.
TW/99-156B	-	-	-	-	-	-	-	-	-	.	.	G	H	.	.	.	.	.	-	-	-	-	-	-	-	-	-	-	-	-	-	-
TW-02-301	.	.	.	.	K	.	.	L	.	.	.	G	.	.	.	.	T	.	.	.		D	.	V	.	.	.	.	.	.	.	.
TW-02-302	.	.	.	.	K	.	.	.	.	.	.	G	.	.	.	.	T	.	.	.		D	.	V	.	.	.	.	.	.	.	.
TW-04-CB8	.	.	.	.	K	.	.	.	.	.	.	G	.	.	.	.	T	.	.	.		D	.	V	.	.	.	.	.	.	.	.
TW/08-02	.	.	.	.	.	.	.	.	.	.	.	G	.	.	.	.	T	.	.	.		D	.	V	.	.	.	.	.	.	.	.

Residues that are identical to the NDV Clone 30 strain are indicated by a dot (.). The hepta repeated HR-A and HR-B (amino acid residues 74 to 110) and C-terminal extension are indicated above the amino acid sequences. Five antigenic sites on HN protein of NDV, including residues 193–201 (site 23), 345–355 (sites 1 and 14) and a C-terminal domain composed of residues 494 (sites 12), 513-521(sites 12 and 2), and 569 (sites 2) are shown [5]. NL/152608/93: APMV-1/ chicken/NL/ 152608/93; Hebei/01/2012: chicken/China/ Hebei/01/2012; Shandong/ 02/2012: chicken/China/Shandong/02/2012; Beijing/01/2012: chicken/China/ Beijing/01/2012

Previous reports showed that LaSota vaccine can be fully protected against challenge by strains from genetic groups VIb, VIg, VIId and IX [16, 21]. As compared to APMV-1/ chicken/NL/152608/93 [21], we found several substitutions in F and HN proteins of shaanxi NDV isolates, such as in the amino acid sequences of F gene with 9I → T, 13P → L, 14 V → M, 16 V → I, 19 V → I, 22 T → I, 23 L → S, 27R → C, 28P → L, 29 T → V, 51S → N, 109S → P, and in the HN gene with 102 T → I, 350H → Y, 518 N → S, and 519 L → S. As compared to XJ-2/97 NDV genotype VIId strains [16], several substitutions in F and HN proteins of shaanxi NDV isolates were found, such as in the amino acid sequences of F gene with 14 V → M, 16 V → I, 19 V → I, 22 T → I, 28P → L, 51S → N, and in the HN gene with 102 T → I, 350H → Y, 518 N → S, and 519 L → S. As compared to JS/1/97/Go NDV genotype VIId strains [16], several substitutions in F and HN proteins of shaanxi NDV isolates were found, such as in the amino acid sequences of F gene with 3S → P, 5P → S, 8R → N, 9I → V, 13P → L, 14 V → M, 16 V → T, 17 T → V, 19 V → I, 20 M → A, 22 T → A, 26I → V, 28P → L, 51S → N, 52 V → I, 71R → K, and 121 V → I as well as in the HN gene with 102 T → I, 347R → G, 350H → Y, 518 N → S, and 519 L → S. These amino acid residue substitutions may change antigenicity of recent shaanxi NDV strains. More recently, it was reported that a substitution at amino acid residue 81 (I to M or V to M) in the hepta repeated regions of HA glycoprotein reduces HA activity [14]. However, similar mutation was not found in the Shaanxi strain. Additionally, examination of the critical amino acid residues 401E, 416R, and 526Y for receptor binding [22] in the HN glycoprotein of recent Shaanxi strains of NDV were also conserved.

### Phylogenetic analysis of F and HN genes of NDV

**Fig. 1:**
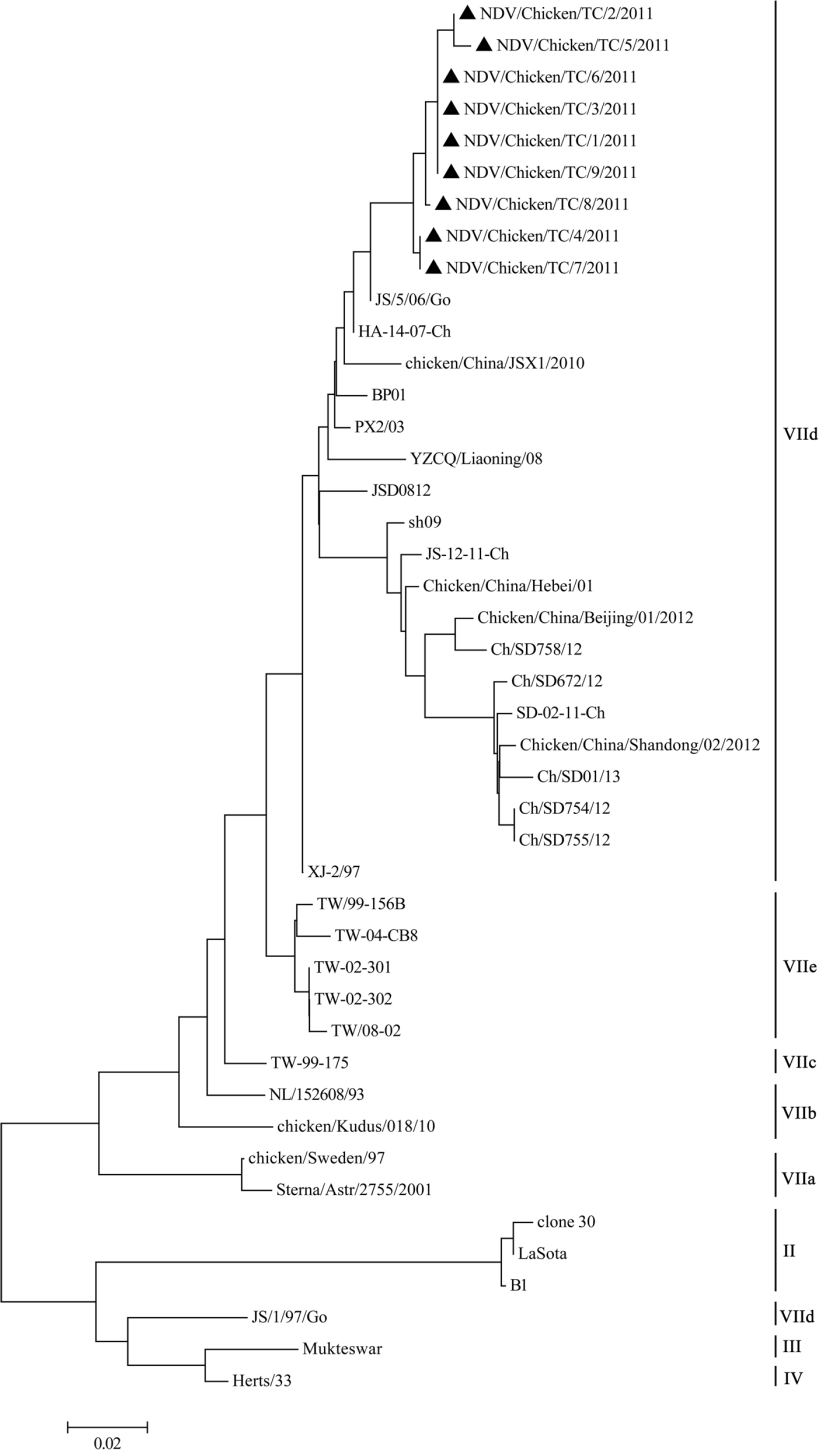
Phylogenetic tree of the nucleotide sequence of NDV strains based on a variable portion (nt 1–389) of the F gene. The accession numbers of the sequences derived in this study and those derived from GenBank were shown in Table 1. The tree was constructed using MEGA 4 neighbor-joining method with 1000 replicates of bootstrap. Genotype, sub-genotype and recently Chinese isolates are indicated on the right. ▲: Shaanxi NDV isolates

Based on the nucleotide sequences (1–389 bp) of the F gene among NDV isolates, a phylogenetic tree was constructed (Fig. 1). The reference strains cover traditional vaccine strains and different pandemic VII genotype NDV strains in China (Fig. 1; Table 1). All recent Shaanxi strains were phylogenetically closely related and belonged to genetype VIId (Fig. 1). Furthermore, all recent Shaanxi strains were not phylogenetically related to vaccine strains, such as Mukteswar, Clone 30, Lasota, B1 and Herts/33. Furthermore, all recent Shaanxi strains were

phylogenetically related to HA-14-07-Ch and JS/5/06/Go NDV strains that were isolated from the Eastern China.

**Fig. 2:**
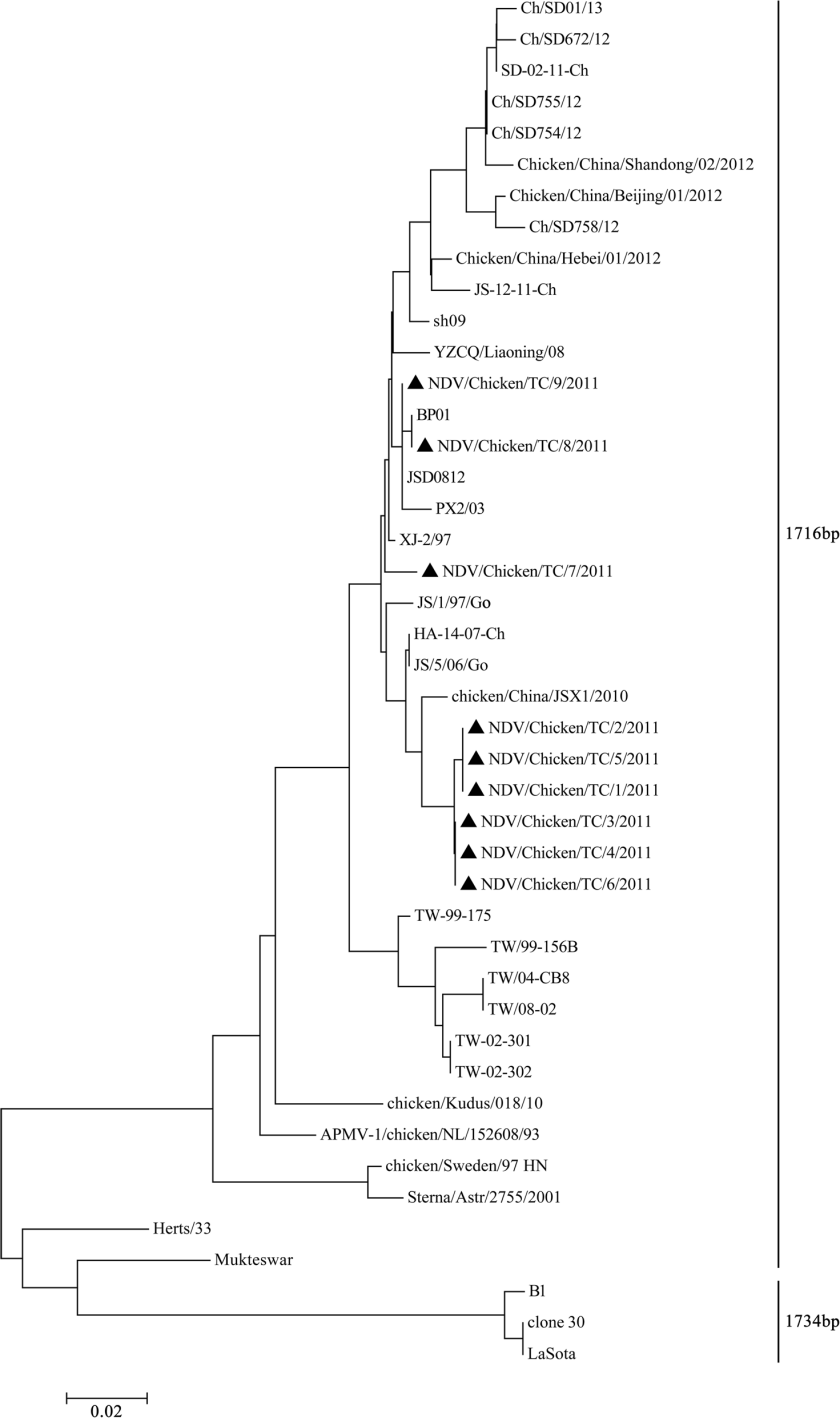
Phylogenetic tree creased on the basis of the full-length HN gene of NDV isolates and reference strains. The accession numbers of the sequences derived in this study and those derived from GenBank were shown in Table 1. The tree was constructed using MEGA 4 neighbor-joining method with 1000 replicates of bootstrap. Genotype, sub-genotype and recently Chinese isolates are indicated on the right. ▲: Shaanxi NDV isolates

Based on nucleotide sequences of the full-length HN gene among NDV isolates, a phylogenetic tree was created (Fig. 2). The results in Fig. 2 showed that all recent Shaanxi strains were phylogenetically closely related and had only 571 amino acid residues. Besides, all these strains were phylogenetically related to chicken/China/ JSX1/2010, HA-14-07-Ch and JS/5/06/Go isolated from the Eastern China. However, all recent Shaanxi strains were not phylogenetically related to vaccine strains. In addition, JS/1/97/Go strain was closely related to the vaccine strain Mukteswar and Herts/33 in the phylogenetic tree based on a variable portion (nt 1–389) of the F gene, but JS/1/97/Go strain was closely related to HA-14-07-Ch in the phylogenetic tree creased on the basis of the full-length HN gene.

### Relatedness (R) values of Shaanxi and LaSota strains

The neutralizing antibody (NA) titers of LaSota and NDV/Chicken/TC/1/2011 vaccination hyperimmune sera were determined by neutralization test. While NDV LaSota was used as an antigen, the NA geometric mean titer (GMT) to both LaSota and NDV/Chicken/TC/1/2011 sera were 128 and 148, respectively. Furthermore, the NA GMT to both LaSota and NDV/Chicken/TC/1/2011 vaccination sera were 67.2 and 241.8, respectively when all nine recent Shannxi strains were used as antigens.

Relatedness (R) values, determined by cross VN, revealed that all the recent Shaanxi isolates are closely related and belonged to a single serotype. However, the cross VN test revealed that R values obtained between NDV/Chicken/TC/1/2011 and LaSota vaccine strain was 0.54, indicating that they were minor subtype difference.

### Hemagglutination inhibition assay

The HI assay was employed to evaluate antibody titers using LaSota antigens. As illustrated in Table 4, the HI titer was undetectable in un-inoculated and negative control group chickens. The chickens exhibited HI titer that received Oil-TC/1 were 7.1 log_2_, received Oil-Las were 7.2 log_2_, received Live-Las were 7.0 log_2_, received Oil-TC/1and Live-Las were 8.1 log_2_, received Oil-Las and Live-Las were 8.7 log_2_.

**Table 4:** The titer of HI antibody of all birds immunized with different vaccines using the LaSota antigen

	Number of chicken												Mean value
Group	1	2	3	4	5	6	7	8	9	10	11	12	
Oil- TC/1	6^a^	8	7	6	8	8	8	8	7	6	6	7	7.1
Oil-Las	7	7	6	9	8	7	7	7	6	7	9	6	7.2
Live-Las	7	7	7	8	7	8	6	7	6	6	7	8	7.0
Oil-TC/1 plus Live-Las	8	8	8	9	8	10	8	7	8	6	8	9	8.1
Oil-Las plus Live-Las	8	8	9	10	8	10	8	9	8	8	9	8	8.7
Un-inoculated control	0	0	0	0	0	0	0	0	0	0	0	0	0
Negative control	0	0	0	0	0	0	-	-	-	-	-	-	0

^a^The titer of HI antibody was expressed on a log _2_ scale. All chickens (*n* = 6) in the negative control group had no clinical signs during the course of the experiment

The cross-HI assay was employed to evaluate the HI antibody titers using different antigens between NDV/Chicken/TC/1/2011 and LaSota. Anti-NDV/Chicken/TC/1/2011 hyperimmune serum was used as an antibody, the HI titer were 9Log_2_ when using the homologous antigens and7Log_2_ when using the vaccine strain LaSota. When anti-NDV LaSota hyperimmune serum was used as an antibody, the HI titer were 10Log_2_ that using the homologous antigens and 7Log_2_ that using NDV/Chicken/TC/1/2011.

### Neutralization test and cross-protectivity experiments

As shown in Table 5, neutralization titers of chicken immunized with different vaccine combinations while NDV/Chicken/TC/1/2011 strain was used as an antigen in neutralization test. The neutralization titer was undetectable in both un-inoculated and negative control group chickens. Our results reveal that chickens having neutralization titers of 64 could be protected against challenge with the NDV/Chicken/TC/1/2011 strain (Table 5).

**Table 5:** Neutralization titres of chicken immunized with different vaccine combinations using NDV/Chicken/TC/1/2011 strain as an antigen

	Serum of chicken											
Vaccine group^a^	1	2	3	4	5	6	7	8	9	10	11	12
Oil-TC/1	128	256	256	64	256	256	256	256	128	64	128	128
Oil-Las	64	64	32^b^	64	64	64	64	64	64	64	128	16^b^
Live-Las	64	64	64	64	64	128	32^b^	64	16^b^	32^b^	64	64
Oil-TC/1 plus Live-Las	64	64	64	128	256	128	128	128	128	256	256	256
Oil-Las plus Live-Las	64	64	128	256	128	128	128	128	32^b^	64	128	128

^a^The neutralization titer was undetectable in both un-inoculated and negative control group chickens

^b^death

To evaluate the protective efficacy of different vaccine combinations, the cross protectivity of each group vaccine was assessed in 3 week after the last vaccine (Table 6). Chickens in the negative control groups had no clinical signs during the course of the experiments. All birds in the un-inoculated control group displayed severe depression and typical lesions from day 3 to 5 pc and 100 % mortality was observed on day 7 pc. Necropsy of these un-inoculated control birds revealed gross lesions consistent with a virulent NDV infection including hemorrhages and edema in the conjunctiva of the lower eyelid, petechial hemorrhages in the thymus, and multifocal hemorrhage of the proventriculus. No obvious clinical signs or mortality was observed in birds receiving either Oil-TC/1 or Oil-TC/1and Live-Las vaccines, and conferred protection from morbidity and death in all chickens subjected to the NDV/Chicken/TC/1/2011 strain challenge (Table 6).

**Table 6:** Antibody response and post-challenge mortality of chicks immunized with different vaccines and then challenged with NDV/Chicken/TC/1/2011

NDV vaccine ^a^	Mortality in each challenge^b^ (%) 7DPC ^c^	NDV-HI GMT^d^ against LaSota strain 56 DPV^e^	14 DPC
Oil-TC/1	0	147	1024
Oil-Las	17	147	1024
Live-Las	25	156	2048
Oil-TC/1 plus Live-Las	0	274	2048
Oil-Las plus Live-Las	9	362	2194
Un-inoculated control	100	All died	All died

^a^At the age of 3 weeks, twelve chicks per group were vaccinated, respectively. After 2 weeks, a booster dose of each vaccine was administered to the bird. Blood samples were taken on 3 week after booster

^b^Oral challenge with the allantonic fluid of virulent NDV/Chicken/TC/1/2011 isolate

^c^DPC: days post-challenge

^d^NDV-HI GMT: geometric mean titer of NDV hemagglutination-inhibition titer

^e^DPV: days post-vaccination

Obvious clinical signs were seen in part of other 3 vaccinated groups by 3 days post-inoculation (dpi). They displayed depressed, consumed less food and water. Two birds died on 5 dpi in Oil-Las group during the experiment, and the mortality rate was 17 %. Three birds died on 5 and 7 dpi in LaSota attenuated vaccines group during the experiment, and the mortality rate was 25 % (Table 6). One birds died on 7 dpi in the Oil-Las and Live-Las vaccines group during the experiment, the mortality rate was 9 %. However, both Oil-Las and LaSota attenuated vaccines conferred protection 83 % and 75 % of chickens, respectively. Furthermore, combination of the Oil-Las and Live-Las vaccine conferred protection to 92 % of chickens challenged with the NDV/Chicken/TC/1/2011 strain (Table 6).

### Frequency of isolation of challenge virus in different vaccine groups

**Fig. 3:**
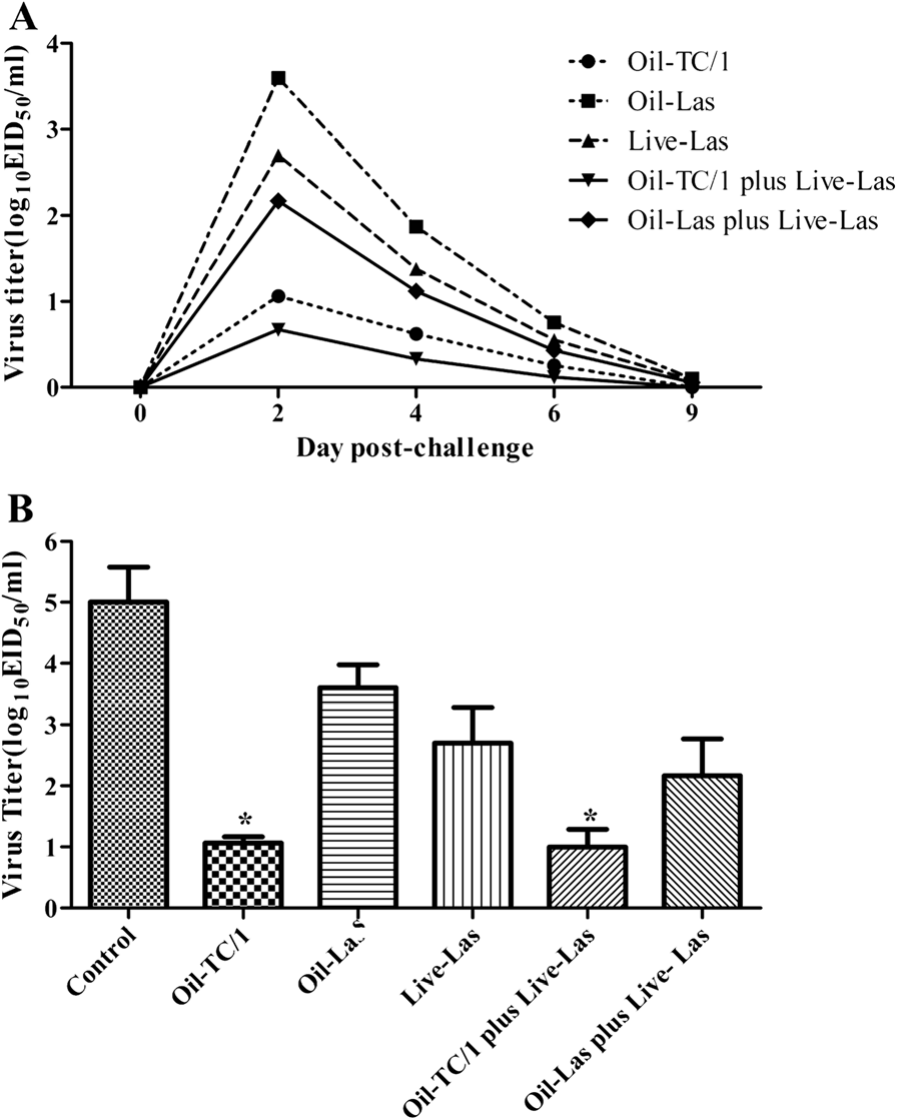
Virus shedding titers from bird oropharyngeal swabs collected on selected days after NDV/chicken/TC/1/2011 strains challenge. **a** Mean virus titers of oropharyngeal swabs of immunized groups on all sampling days. **b** Comparison of oropharyngeal virus titers at 2 day post-challenge. *Significant difference (P < 0.05) from Oil-Las

Virus shedding was examined in all experimental chickens on day 0, 2, 4, 6, and 9 pc. As shown in Table 7, viruses could be detected in both oropharyngeal and cloacal swabs in challenge un-inoculated control group from day 2 pc. No virus was isolated from any bird in the negative control group. On day 4 pc, there were 83.3 %, 75 % and 66.7 % positive oropharyngeal swabs could be detected respectively from the Oil-Las vaccines group, Live-Las vaccines group and the vaccines group with Oil-Las and Live-Las. In this study, chickens immunized with an Oil-TC/1 vaccine have lower viruses shedding in day 2 pc from oropharyngeal samples than those immunized with a Live-Las vaccine (Fig. 3a). The virus titers of oropharyngeal swabs from both Oil-TC/1- and Oil-TC/1 plus Live-Las-inoculated chickens were significantly reduced compared to the titers of three other vaccinated groups as well as the un-inoculated control (Fig. 3b).

**Table 7:** Frequency of detection of challenge virus in different vaccine groups

Group	Post-challenge samples (no. positive/total)							
	Day 2		Day 4		Day6		Day9	
	O^a^	C^b^	O	C	O	C	O	C
Oil- TC/1	12/12^c^	10/12	4/12	4/12	2/12	1/12	0	0
Oil-Las	12/12	11/12	10/12	8/12	7/10	6/10	3/10	2/10
Live-Las	12/12	10/12	9/12	8/12	5/10	4/10	2/9	2/9
Oil- TC/1plus Live-Las	12/12	10/12	3/12*	3/12*	1/12	0	0	0
Oil-Las plus Live-Las	12/12	11/12	8/12	7/12	5/12	3/12	2/11	1/11
Un-inoculated control	12/12	12/12	12/12	12/12	NS^d^	NS	NS	NS

^a^Oropharyngeal swabs. ^b^Cloacal swabs. ^c^The number in front of the slash represents the number of animals, which had detectable viral genome RNA; the number behind the slash stands for the total number of animals in this group. ^d^No survivors. *Significant difference (P < 0.05) among immunized groups with the column

## Discussion

There have been at least four major outbreaks of Newcastle disease worldwide so far and every epidemic had its specific genotypes [4, 23]. The NDVs currently circulating worldwide exhibit multiple genotypes. Outbreaks of Newcastle disease in vaccinated chicken flocks have been reported previously in China and even in Taiwan [2, 3, 5, 24]. Velogenic NDV strains were isolated from egg layer flocks with NDV vaccine immunization failure in China [24, 25]. To date, the genotype VII of NDVs is predominant in the domestic poultry of Asia. The sub-genotypes VIIc and VIId of NDVs existed in China and Far East countries [20, 24, 26], while sub-genotype VIIe of NDVs was found in Taiwan [5]. Earlier studies have suggested that high genetic diversity and multiple genotypes of NDVs in China were found [17, 19, 24, 27]. Sub-genotypes VIIc andVIId of NDVs were the major epidemic strains in China in recent years and could evolve into other NDV genotypes [24].

Analysis of the deduced amino acid sequences of the F protein of recent Shaanxi strains revealed that all these isolates possessed the amino acid sequences ^112^R-R-Q-K-R-F^117^ for the C-terminus of the F2 protein, indicating that they were all highly pathogenic viruses. The result was consistent with the pathogenicity assay. Additionally, all the recent Shaanxi strains had 101 K and 121 V, a characteristic of genotype VII viruses [15]. Furthermore, phylogenetic analysis indicated that all Shaanxi strains were classified as sub-genotype VIId. Analysis of deduced amino acid sequences of the F protein of recent Shannxi and traditional vaccine strains revealed many amino acid residue substitutions. It is worth to note that many amino acid residue substitutions on F protein of recent Shannxi NDV strains were also found as compared to the pandemic strains in China during the past 10 years. The findings suggest that these mutations on the F proteins may change antigenicity of recent Shaanxi NDV strains.

Earlier studies have demonstrated that the viral HN protein mediates attachment to sialic acid-containing receptor(s) via its neuraminidase (NA) activity, the apparently opposing activity of release of sialic acid from soluble and membrane-associated glycoconjugates [28–31]. It was also suggested that there are at least 5 antigenic sites related to neutralizing epitopes on the HN protein of NDV, such as residues 193 to 201 (site 23), residues 345 to 355 (sites 1 and 14) and a C-terminal domain composed of residues 494, 513 to 521, and 569 (sites 12 and 2). Furthermore, monoclonal antibodies (MAbs) to 3 overlapping sites (2, 12, and 23) on HN glycoprotein suppressed the NA activity of the virus [32, 33]. MAbs to sites (1 and 14) inhibited HA predominantly by preventing viral attachment to chick cells [32, 34]. As compared to vaccine and the past pandemic strains in China, recent Shaanxi NDV strains reveal a number of amino acid residue substitutions at neutralizing epitopes on HN protein, which suggest that these mutations may lead to antigenic change and have effect on viral attachment to the receptor on the cell surface [33, 35]. This divergence further elucidates that amino acid residue substitutions may lead to the change of antibody recognition capabilities and types. Apparently, some new immune response-escaping antigenic variants were developed and resulted in ND outbreaks in Shannxi province in China. In this work, an inactivated oil mulsion (Oil-TC/1) vaccine prepared from a recent Shannxi isolate (NDV/Chicken/TC/1/2011), conferred protection from morbidity and death in all chicks subjected to homologous challenge whereas the inactivated oil mulsion Lasota vaccine (Oil-Las) and LaSota attenuated vaccine (Live-Las) conferred clinical protection to only 83 % and 75 % of chicks, respectively challenged with NDV/Chicken/TC/1/2011. It can be speculated that immunization failure may be caused by the difference of antigenic sites between vaccine strains and field strains.

In an earlier study, Liu and his colleagues also showed that SPF chickens vaccinated with LaSota vaccines could be fully protected against challenge by strains from genetic groups VIb, VIg, VIId, and IX [16]. Recently, Cornax et al. reported LaSota vaccine could provide full protection for NDV genotype VIId [21]. Conversely, several reports in using LaSota vaccine to immunize chicken in China or Taiwan [5, 25] demonstrated that LaSota vaccine could not provide full protection for genotype VII of NDV. In the present study, LaSota vaccine was not capable of providing full protection for the flocks. As compared to APMV-1/chicken/NL/152608/93, XJ-2/97, and JS/1/97/Go NDV strains of genotypes VIId and VIIb [16, 21], we found several substitutions in both F and HN proteins of shaanxi NDV isolates, thereby altering antigenicity of recent shaanxi NDV strains.

Vaccination is the most important way to prevent NDV infections [16, 24]. In the present study, the inactivated vaccine prepared from a recent Shaanxi strain NDV/Chicken/TC/1/2011 protected all the chickens from morbidity and mortality against its homologous challenge. However, the group immunized with LaSota strain inactivated and attenuated LaSota vaccines had 17 % and 25 % mortality rate, respectively. Our results are argreement with previous reports in using LaSota vaccine to immunize chicken in China or Taiwan [5, 25]. Furthermore, no obvious clinical signs or mortality was observed in birds receiving either Oil-NDV/Chicken/TC/1/2011 or Oil-NDV/Chicken/TC/1/2011 plus attenuated LaSota vaccines, and conferred protection from morbidity and death in all chickens subjected to NDV/Chicken/TC/1/2011 strain challenge.

## Conclusions

In conclusion, this study provides evidences, suggesting that recent Shaanxi strains are immune response-escaping antigenic variants that were responsible for these new NDV outbreaks in northwestern China. Therefore, a ND inactivated vaccine must be prepared from new local strains of velogenic NDV.

## Materials and methods

### Virus isolation and animals

Forty Newcastle diseases suspected lungs; tracheal rings of the dead chickens (either Hy-Line Brown or Roman White chickens) from nine different poultry farms were collected from both Tongchuan city and Yaoxian county of Shaanxi province in China during 2011. The ages of these chickens range from 9 to 38–week-old. All samples were propagated in the allantoic sacs of 10-day-old SPF chicken embrocated eggs at 37 °C for 3–7 days. Hemagglutination (HA) tests and hemagglutination-inhibition (HI) tests were conducted as described previously [36]. Nine NDV strains were purified by plaque purification three times. Each virus was coded as follows: virus name/isolation host/isolation place abbreviation/case number/year isolated. For example, NDV/Chicken/TC/1/2011 indicates that the sample is case no. 1 of a NDV strain isolated from the 2011 outbreak in Tongchuan, Shaanxi province. Intracerebral pathogenicity index (ICPI) test and mean death time (MDT) of chicken embryos test were performed using the OIE recommended method. All NDV isolates and reference isolates are shown in Table 1.

Seventy-eight of 5 days old specific pathogen-free (SPF) White Leghorn chickens were supplied by Green Square Biological Engineering Company, Yangling, china. All birds were housed in isolators in the laboratory animal facility under negative pressure with food and water provided ad libitum. At the age of 3 weeks, the birds were inoculated with vaccines. All animal experimental procedures were approved by the Ethical and Animal Welfare Committee of Shannxi Province and were in compliance with the China law on animal experiments.

### Viral RNA extraction for reverse transcription (RT)-polymerase chain reaction (PCR)

Viral RNA was extracted using TIAN amp Virus DNA/RNA kit according to manufacturer instructions. To perform RT-PCR, the NDV-RNA was extracted directly from the allantoic fluid of the NDV-inoculated embryos. After purification, the purified NDV genomic RNA was used to synthesize cDNA by reverse transcription. The primers for amplification of the F and HN genes of NDV were designed on the basis of LaSota sequences (accession no. JF950510). The primer sequences for amplification of F gene were F1 (5′-TGTAGTAACGGGAGACAAAG CAG-3′; identical to nucleotides 4669 to 4691) and F2 (5′-GAATAAATACCAG AGACATAGGGA-3′; complementary to nucleotides 5625 to 5601). The expected size of a PCR-amplified fragment is 957 bp in length. The upstream region of the HN gene of NDV isolates was amplified by PCR with the primers: HN1 (5′-AATTTCATCCCAGCGCCTAC-3′, identical to nucleotides 6893 to 6912**)** and HN2 (5′-ATCATCAAGCATCGTCCC-3′; complementary to nucleotides 7894 to 7877**)**. Primers HN3 (5′-AGAGTCGTGCTGGAGAAT-3′, identical to nucleotides 6431 to 6448**)** and HN4 (5′-CTACCTGTCCGAGTGAAA-3′; complementary to nucleotides 7768 to 7751) were used to amplify the downstream region of the HN gene. The expected upstream and downstream regions of the PCR products are 1002 bp and 1338 bp in length, respectively. These 2 regions overlap and cover the full-length HN gene. The synthesized cDNA was used as the template for PCR amplification. PCR reactions of F gene were subjected to 35 cycles consisting of denaturation for 50 s at 94 °C, annealing for 50 s at 49 °C, and extension for 1 min at 72 °C and one final extension cycle at 72 °C for 10 min. The annealing temperatures of HN1 and HN2 genes were 43 °C and 46 °C, respectively, and other procedures were same as described above. After completing PCR, 10 μL of the reaction mixture was loaded onto a 1.5 % agarose gel containing 0.5 μg/mL of ethidium bromide for electrophoresis and subsequent visualization by UV transillumation. The PCR products were sent out for sequencing after cloning purified.

### Amino acid sequence and phylogenetic analyses

The F and HN gene sequences of NDVs were analyzed and compared by a DNAStar software program. Nucleotide and deduced amino acid sequences of emerging Shaanxi strains of NDV were compared with the previously published sequences. Clustalx and Mega 4.0 were used to construct phylogenetic trees based on the F (1–389 bp) and the full-length HN gene (1716 bp) sequences. All recent Shaanxi strains, vaccine strains, and reference NDV strains were listed in Table 1. The Clone 30, LaSota, Mukteswar, and B1 strains are attenuated vaccine strains that widely used in China and in the world. The Herts/33 is an inactivated vaccine strain.

### Hemagglutination inhibition assay

Blood samples were taken on 3 week after the last vaccination, and sera were isolated and the hemagglutination inhibition (HI) assay was performed using four haemagglutinating units of antigen. Sera of un-inoculated birds were collected and used as a negative control. All sera were inactivated at 56°Cfor 30 min and stored at −80 °C until used. The geometric mean antibody titers were expressed on a log_2_ scale. The antigen used in this experiment was LaSota vaccine strain that is used as a standard antigen in the HI test routinely used in ND diagnostic laboratories.

### Cross virus-neutralization (VN) assay

The serologic relatedness of LaSota and NDV/Chicken/TC/1/2011 NDV strains as well as between NDV/Chicken/TC/1/2011 strain and other eight Shannxi strains were determined using a virus-neutralization (VN) test. The cross-reactivity was examined and R value was determined as described previously [37]. R value is to determine the degree of antigenic similarity of two viruses, which can be used for the similarity analysis of the protein antigen in different virus strains. Ten groups of 30 specific-pathogen-free broilers each were first vaccinated with either inactivated vaccines (nine Shannxi NDV strains) or LaSota vaccine, and then boosted by either Shannxi NDV strains or LaSota vaccine. Serum was reacted against each other in a VN test. Briefly, Two-fold serial dilutions of sera were mixed with approximately 100 tissue culture infectious doses (TCID) 50 % for 60 min at 37 °C in 96-well microtiter plates. The TCID_50_ of nine Shannxi NDV strains and LaSota vaccine strain were measured by the method of Reed and Muench on chicken embryo fibroblast (CEF). After the hyperimmune serum of two viruses were 2-fold serially diluted, they were mixed with equal volume containing 100 TCID_50_ two viruses and then reacted at room temperature for 1 h. The culture medium was discarded and virus-serum mixture was added to trace cell reaction plates containing monolayer cells as 0.1 ml/well. Each dilution was carried out for 8 repeats. Addition of maintenance medium after 37 °C for 2 h, while virus control containing 100 TCID_50_, blank cell control and serum control were included. Calculation of the 50 % serum neutralization end after culturing 72 h was performed. All neutralization tests were performed twice and geometric mean titer (GMT) were calculated. The *R*-value was calculated as described previously [38]. *R* values higher than 0.70 prove antigenic identity, *R* values between 0.70 and 0.33 prove antigenic relatedness meaning minor subtype differences, and *R* values between 0.32 and 0.11 indicate loose relatedness meaning major subtype difference, whereas *R* values below 0.11 indicate no relatedness at all meaning serotype difference [37].

### Cross-protectivity

Both NDV/Chicken/TC/1/2011 strains and vaccine strain LaSota were used to prepare monovalent oil-emulsion vaccines as described previously [5]. Live vaccine of the LaSota strain from a commercial source (Green Square Biological Engineering Company, Yangling, china) was also used. As shown in Table 8, seventy-eight SPF White Leghorn chickens were randomly divided into seven groups. At the age of 3 weeks, the chickens were inoculated. Birds received LaSota attenuated vaccine viruses that were inoculated one dose commercial live-LaSota (Live-Las) via eye-drop and intra-nasal routes. Birds received inactivated vaccines that were injected subcutaneously with 0.4 ml of inactivated oil emulsion-NDV/Chicken/TC/1/2011 strains (Oil-TC/1) and oil emulsion-LaSota (Oil-Las), respectively, while the control group was injected with phosphate-buffered saline (PBS). After 2 weeks, a booster dose of each vaccine was administered to the birds.

**Table 8:** Experimental groups and design for chickens used in this study

Group	Number of chicks	Vaccine dose	Challenged virus strain and dose
Oil-TC/1	12	0.4 ml	10^6^EID_50_ NDV/TC/1^a^
Oil-Las	12	0.4 ml	10^6^EID_50_ NDV/TC/1
Live-Las	12	1 dose	10^6^EID_50_ NDV/TC/1
Oil-TC/1 plus Live-Las	12	0.4 ml/1 dose	10^6^EID_50_ NDV/TC/1
Oil-Las plus Live-Las	12	0.4 ml/1 dose	10^6^EID_50_ NDV/TC/1
Un-inoculated control	12	PBS	10^6^EID_50_ NDV/TC/1
Negative control	6	PBS	PBS

^a^ NDV/TC/1: NDV/Chicken/TC/1/2011; Oil-TC/1: inactivated oil mulsion-NDV/ Chicken/TC/1/2011; Oil-Las: oil emulsion-LaSota; Live-Las: LaSota attenuated vaccine

The cross protectivity of each group vaccine was assessed in 3 week after booster. All birds were challenged through eye-drop and intra-nasal routes with 10^4^ ELD_50_ of NDV strain NDV/Chicken/TC/1/2011. Challenge-free birds were administrated with PBS via the same route as the negative control. Following challenge, birds were observed for clinical signs and death during 14 day post-challenge (pc). Moribund chickens were euthanized with intravenous sodium pentobarbital at a dose of 100 mg/kg and counted dead for the next day.

Necropsies were completed on selected birds to assess the presence of gross pathological lesions. Oropharyngeal and cloacal swabs were collected at 0, 2, 4, 6 and 9 days pc for virus isolation and titration as previously described [39] and virus titers were expressed as log_10_ EID_50_/ml. The oropharyngeal positive samples were then quantified the viral loads as previously reported [4].

### Statistical analysis

Statistical analysis of serology titers and virus titers were performed using IBM statistical package for social sciences (SPSS) statistical software. A probability (p) value < 0.05 was considered statistically significant.
